# Deep Learning‐Enabled STEM Imaging for Precise Single‐Molecule Identification in Zeolite Structures

**DOI:** 10.1002/advs.202408629

**Published:** 2024-12-20

**Authors:** Yaotian Yang, Hao Xiong, Zirong Wu, Zhiyao Luo, Xiao Chen, Xiaonan Wang, Fei Wei

**Affiliations:** ^1^ Department of Chemical Engineering Tsinghua University Beijing 100084 China; ^2^ Ordos Laboratory Ordos Inner Mongolia 017000 China

**Keywords:** deep learning, low‐dose STEM Image, single‐molecule detection and analysis

## Abstract

Observing chemical reactions in complex structures such as zeolites involves a major challenge in precisely capturing single‐molecule behavior at ultra‐high spatial resolutions. To address this, a sophisticated deep learning framework tailored has been developed for integrated Differential Phase Contrast Scanning Transmission Electron Microscopy (iDPC‐STEM) imaging under low‐dose conditions. The framework utilizes a denoising super‐resolution model (Denoising Inference Variational Autoencoder Super‐Resolution (DIVAESR)) to effectively mitigate shot noise and thereby obtain substantially clearer atomic‐resolved iDPC‐STEM images. It supports advanced single‐molecule detection and analysis, such as conformation matching and elemental clustering, by incorporating object detection and Density Functional Theory (DFT) configurational matching for precise molecular analysis. the model's performance is demonstrated with a significant improvement in standard image quality evaluation metrics including Peak Signal‐to‐Noise Ratio (PSNR) and Structural Similarity Index Measure (SSIM). The test conducted using synthetic datasets shows its robustness and extended applicability to real iDPC‐STEM images, highlighting its potential in elucidating dynamic behaviors of single molecules in real space. This study lays a critical groundwork for the advancement of deep learning applications within electron microscopy, particularly in unraveling chemical dynamics through precise material characterization and analysis.

## Graphical abstract

An innovative deep learning framework utilizes a two‐stage DIVAESR model based on noise reconstruction and detailed enhancement incorporating domain knowledge to achieve atomic‐level clarity in low‐dose iDPC‐STEM images. This approach significantly optimizes shot noise handling and enables precise single‐molecule detection and analysis in complex structures, highlighting its potential for real‐time elucidation of single‐molecule dynamics.

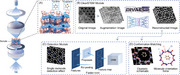

## 1 Introduction

Scanning Transmission Electron Microscopy (STEM) is recognized as a powerful tool in scientific research, with various imaging modes achieving sub‐angstrom spatial resolution and theoretically capable of observing atomic‐level dynamic behaviors in real‐time.^[^ ^1^ ^]^ It plays an indispensable role in studying the properties of materials and chemical processes.^[^ ^2^ ^]^ Traditional STEM imaging, however, relied on annular detectors to receive scattered electrons at high angles, which suffer from low electron utilization efficiency and necessitate high electron doses (>10^6^ e^−^ Å^−2^), leading to beam damage and artifacts in beam‐sensitive materials such as zeolites, Metal‐Organic Frameworks (MOFs), perovskites, etc.^[^ ^3^ ^]^

High‐Resolution Transmission Electron Microscopy (HRTEM) and Annular Dark‐Field STEM (ADF‐STEM) are utilized to reveal atomic‐scale local structural information within zeolite catalysts, including surfaces, interfaces, defects, and dopants.^[^ ^4^ ^]^ These advanced imaging methods, integrated with other spectroscopic techniques, enhance our understanding of the structure‐to‐performance relationship of zeolite catalysts and lay scientific foundations for designing more efficient catalytic systems. Simultaneously, recent advancements in iDPC‐STEM have significantly enhanced electron utilization efficiency through the use of segmented annular detectors positioned at lower angles (4–22 mrad), enabling efficient low‐dose imaging.^[^ ^1^, ^3^, ^5^ ^]^ Moreover, it offers imaging contrast with a near‐linear relationship to atomic numbers, facilitating the simultaneous imaging of both light and heavy elements. Such advancements have enabled atomic‐level observation of single molecules' dynamic behaviors within zeolite pores, highlighting how local environments and acidic site distributions affect molecular adsorption configurations.^[^ ^1^, ^2^, ^6^ ^]^ However, iDPC‐STEM is not without its limitations under low electron dose conditions, as the significant amount of shot noise complicates the precise identification of single‐molecule positions and conformations. Additionally, the analysis and quantification using these electron microscopy techniques often require a significant amount of time and expertise from professionals, which limits their broader application and underscores the urgent need for advanced analytical techniques that move beyond traditional, time‐consuming, expert‐dependent manual interpretations.^[^ ^7^ ^]^

The advent of deep learning has heralded revolutionary methods for enhancing iDPC‐STEM images,^[^ ^7^, ^8^ ^]^ outperforming traditional denoising approaches, such as Principal Component Analysis (PCA) and Independent Component Analysis (ICA).^[^ ^7^, ^9^ ^]^ Machine learning image recognition methods,^[^ ^10^ ^]^ honed on extensive datasets, exhibit superior proficiency in addressing the subtleties of image variations, thereby excelling in areas of atomic localization, single‐atom dynamics, defect identification, and image denoising and enhancement.^[^ ^8^, ^11^ ^]^ Despite significant advancements in STEM imaging, the application of deep learning under low‐dose conditions has largely focused on denoising and enhancing images with clearly structural frameworks.^[^ ^11^, ^12^ ^]^ The full potential of deep learning for complex and finely detailed structures remains largely untapped. This gap highlights the urgent need for more sophisticated algorithms capable of adapting to challenging imaging conditions. Additionally, the extended time required to acquire specific iDPC‐STEM images limits data availability, posing substantial challenges to the broad application of deep learning in electron microscopy imaging.

In this work, we initially conducted a comparative analysis of several classic models. The results of these methods, applied directly to low‐dose STEM images, are detailed in Extended **Table** **1**. Based on the limitations of these approaches, we developed a cutting‐edge artificial intelligence (AI)‐driven framework, specifically optimized and enhanced for processing low‐dose iDPC‐STEM images. At its core is a novel denoising enhancement model, the DIVAESR, engineered specifically for low‐dose iDPC‐STEM imaging. This model significantly improves the quality of obtained images, serving as an effective tool for subsequent single molecule detection and molecular conformation matching analysis through the use of the object detection model,Faster‐RCNN.^[^ ^10a^ ^]^ Considering the scarcity and high cost of real datasets, we generated simulated iDPC‐STEM images with various noise characteristics using the abTEM software under expert guidance for the training phase of our model. During testing, we applied our framework to actual, imperfect iDPC‐STEM images, showcasing its outstanding transfer learning capabilities. Our method significantly enhances the detection of weak single‐molecule signals in electron microscopy, providing substantial improvements and novel insights into the imaging of electron beam‐sensitive materials.

## 2 Results and Discussion

### 2.1 Advanced Framework for Enhanced iDPC‐STEM Imaging in Single Molecule Detection and Analysis

**Figure 1:**
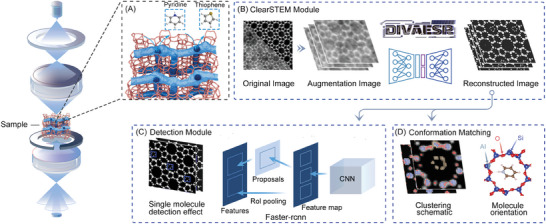
Advanced Framework for Enhanced STEM Imaging in Single Molecule Detection and Analysis. The process first generates simulated data and acquires experimental images, then denoises and enhances low‐dose noisy images through the DIVAESR model, with the resulting high‐quality, clear images further undergoing single‐molecule point detection and analysis. A) The generation of simulated iDPC‐STEM images using abTEM. B) Robust data augmentation techniques to ensure the model's reliability. The DIVAESR model denoises noisy images for the next procedure. C) Single‐molecule (thiophene and pyridine) detection. D) Clustering element analysis and DFT optimal conformation matching for Al site localization.

The framework employs the DIVAESR model for noise reduction to enhance the original STEM images, resulting in clearer pictures for analysis. This facilitates subsequent single molecule detection (such as pyridine and thiophene) using Faster R‐CNN, as well as optimal conformation matching and elemental clustering analysis, as illustrated in **Figure** **1**.

In Figure 1, module (A) and (B), the framework employs the open‐source abTEM software to generate simulated iDPC‐STEM images, which serve as the foundational dataset for training subsequent machine learning models.^[^ ^13^ ^]^ These images are produced using the multi‐slice method, incorporating thermal vibrations and varying shot noise levels based on the Poisson distribution, and are convolved with a Gaussian to mimic the effects of a finite source size, ensuring that the simulation parameters match experimental values (Detailed information on simulated data generation can be found in Materials and Methods, Section S1, Supporting Information: abTEM simulation of iDPC‐STEM images). To enhance the robustness and generalizability of our model, we implemented a series of data augmentation techniques including brightness adjustment, geometric transformations, cropping, and scaling to simulate various conditions that may be encountered in STEM imaging, ensuring that our model can accurately identify molecular structures under a broader range of imaging conditions (Further details can be found in Materials and Methods, Section S1, Supporting Information: Data Augmentation). Augmented images are then fed into the DIVAESR model for training to achieve noise reduction enhancement of iDPC‐STEM images.

As shown in the Detection Module (Figure 1, module (C)), this module uses the Faster R‐CNN model for single molecule detection. The input is the high‐quality, denoised, and enhanced images processed by ClearSTEM Module (Figure 1. module B)), which are used to detect and identify the positions of single molecules of thiophene and pyridine.

As demonstrated in Conformation Matching (Figure 1, module (D)), this module employs clustering and pixel value constraint techniques for element type and small molecule site analysis. After detecting small molecules in the Detection Module, further image conformation matching and aluminum (Al) acid site localization are conducted. Utilizing DFT to calculate optimized molecular conformations of pyridine or thiophene molecules with different Al locations, the most similar conformation pairing is then selected through image matching to infer the corresponding Al sites.

### 2.2 Noise Reconstruction and Super‐Resolution for Precise Single‐Molecule Detection

**Figure 2:**
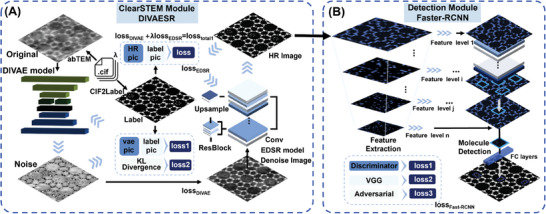
Schematics of the ensemble model include generating electron microscope images and CIF files with abTEM, denoising and enhancing resolution with DIVAESR models, and identifying single molecule sites with Fast‐RCNN. A) ClearSTEM module: DIVAESR model framework. B) Detection module: Faster‐RCNN model framework.

The process is composed of two modules: initially, a preprocessing module for iDPC‐STEM images aimed at noise reduction and image enhancement, followed by a detection module utilizing Faster R‐CNN for precise localization of single molecules, as illustrated in **Figure** **2**.

In the ClearSTEM module (Figure 2A), to achieve clearer denoised and enhanced images, we have developed a new denoising enhancement model, DIVAESR, consisting of two components. Initially, the DIVAE component, inspired by the VAE model, learns from the noise and reconstructs the Noise from the original image, then uses the original image minus the noise to obtain the Denoise image (see Figure S1, Supporting Information for details). However, due to the intrinsic limitations of DIVAE, which involves transformation from lower to higher dimensions leading to information loss, the denoised images lack fine details. Therefore, we feed the denoised images into the SR (Super‐Resolution) component to further enhance the image resolution and refine image details (For more details on the model process, refer to Figure S2, Supporting Information). Our model's innovation also lies in the design of its loss function, composed of two parts. The first part reconstructs noise by differentiating between actual molecular structures and noise artifacts through variational learning. The second part, the SR loss, enhances the details in the reconstructed image by minimizing the loss between the upsampled reconstructed image and the ideal image. By integrating these two loss components into a final loss function, we introduced a factor λ to balance their weights. The impact of varying λ on the loss is detailed in Extended **Table** **2**, where we identified 0.1 as the optimal value. (For more details, see Materials and Methods, Section S1, Supporting Information: DIVAESR Loss Function). The synergistic effect of these two enhancement stages produces high‐resolution images with significantly reduced noise, which is crucial for precise molecule detection. Furthermore, the framework undergoes meticulous iterative training. This training is vital to ensure that the produced images retain essential molecular details, critical for their effective use in advanced scientific analysis. More details can be found in Materials and Methods, Section S1, Supporting Information: DIVAESR Model Training.

Following sophisticated denoising and reconstruction in the ClearSTEM module, the Faster‐RCNN model is adopted, as an advanced visual recognition technology typically used for object recognition and localization in images, such as detecting molecular chirality in Scanning Probe Microscopy^[^ ^10b^ ^]^ (shown in Figure 2B, Supporting Information). The model exploits the noise‐reduced, high‐resolution images to accurately locate and identify individual molecules, despite the challenges presented by varying electron doses in STEM imaging. For more details, see Materials and Methods, Section S1, Supporting Information: Faster‐RCNN Model Training.

The model trained on simulated datasets was also tested on real iDPC‐STEM images. The transferred model was capable of detecting reconstructed positions in critical areas and demonstrated good robustness and generalization compared to other classical models (Figures S11 and S14, Supporting Information). This further indicates that our model has effective denoising enhancement and detection capabilities for more diverse and realistic datasets.

### 2.3 ClearSTEM Module's Superior Image Enhancement Performance

**Figure 3:**
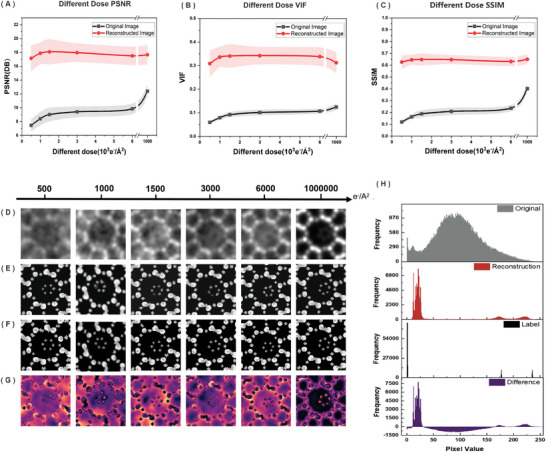
ClearSTEM module Performance Evaluation and Image Enhancement. Comparison between original versus reconstructed images at a variety of electron doses of A) PSNR, B) VIF, and C) SSIM, D–G) Original, reconstructed, labeled images and difference between original and reconstruction at different doses. H) Pixel intensity distribution in original versus reconstructed iDPC‐STEM images.

To assess the enhancements in image clarity and detail provided by our DIVAESR model, we evaluated the reconstructed images using widely recognized image quality metrics. Specifically, we compared the Peak Signal‐to‐Noise Ratio (PSNR), Visual Information Fidelity (VIF), and Structural Similarity Index (SSIM) of the original images against those processed by DIVAESR across various electron doses. As the electron dose increased, error band graphs of PSNR (**Figure** **3**A), VIF (Figure 3B), and SSIM (Figure 3C) distinctly illustrated significant disparities between original (gray) and reconstructed images (red). Notably, the average SSIM for original images increased from 0.13 to 0.38, while the reconstructed images consistently exhibited an SSIM of 0.62, significantly surpassing the original. The PSNR and VIF metrics further affirmed the superior performance of reconstructed images, validating the DIVAESR model's ability to enhance image quality. The shaded areas around the lines represent standard deviation bands, indicating the variability and uncertainty of the measurements.

Visual comparisons of sampled images at different doses were also conducted to illustrate the disparities before and after reconstruction. As shown in Figure 3D,E, the reconstructed images present contrasting effects at varying noise levels, with Figure 3F depicting the corresponding simulation STEM labels. Heat maps (Figure 3G) visualized the differences between the images pre‐ and post‐reconstruction, highlighting the model's significant noise reduction and enhancement capabilities in high‐noise regions, thus revealing details of the optimization process. The histogram analysis (Figure 3H) illustrates the pixel value distributions for the original, reconstructed, and labeled images, highlighting the intensity differences between the original and reconstructed imagery. The model's reconstruction reveals a distinct and enhanced pixel intensity profile, markedly closer to the label's distribution than the flat and uniform distribution of the original images. This contrast underscores the model's effectiveness in noise reduction as well as amplifying key image details, preserving and accentuating the molecular structures within the images.

Considering the evaluation results from SSIM and PSNR, along with the detailed image analyses via histograms and differences, the first stage DIVAESR model exhibited exceptional performance in image reconstruction. Despite some observed differences in regions of high signal‐to‐noise ratio, the model overall successfully enhanced image quality, providing a clearer basis for subsequent molecular identification and localization. These results affirm the effectiveness and significant potential of our model in processing iDPC‐STEM images.

### 2.4 High‐Performance Detection Module Across Multiple Conditions

**Figure 4:**
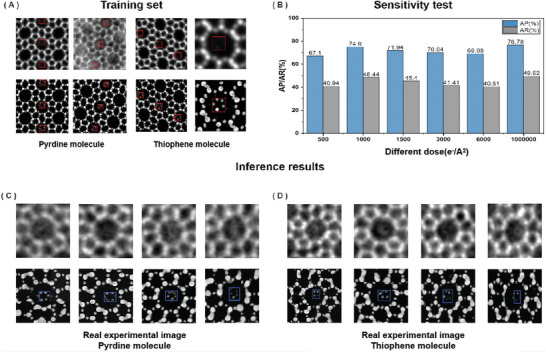
Detection module performance and robustness against dose change analysis. A) Original simulated iDPC‐STEM images of thiophene and pyridine molecular systems from the training set, alongside their corresponding labels, were used to train the Faster R‐CNN model. The molecular structures of thiophene and pyridine molecules are presented. B) The model's performance was tested on the test set under iDPC‐STEM images with varying conditions of electron noise dosage. C,D) Model testing results on real iDPC‐STEM images for pyridine (C) and thiophene (D) Molecules.

In the detection module of our framework, we focused on its ability to accurately detect single molecules under varying levels of electron dosage. During the training phase, simulated iDPC‐STEM images of two different single molecules, marked with their respective positions as shown in **Figure** **4**A, were used. We then evaluated the model's detection effectiveness on iDPC‐STEM images at different doses, by inputting the images enhanced by the ClearSTEM module into the detection module. The detection metrics, Average Precision (AP) and Average Recall (AR) as described in Figure 4B, which are commonly used in object detection, were employed to quantitatively assess the accuracy and precision of the bounding box and classification results. The results showed that the model achieved significant effectiveness in detecting single molecules, with AP rates generally remaining above 70% and AR rates also above 40%.

The inference results further underscored the model's detection capability under various conditions and environments. We tested the performance under multiple conditions including low dose, high dose, high resolution, and low resolution for Pyridine and Thiophene molecules, as well as in simulated (Figures S3 and S4, Supporting Information) and real iDPC‐STEM images. The model demonstrated good detection results for Thiophene (Figure 4C) and Pyridine (Figure 4D) molecules in real iDPC‐STEM images, proving the model's resilience to noise and low image contrast, as well as its feasibility in transitioning from simulated to real scenarios. This consistent performance across different conditions attests to the robustness of the model's feature extraction and target detection mechanisms.

### 2.5 DIVAESR Model‐Assisted Single Molecule Conformation Matching and Determination of Al Sites in Zeolites

**Figure 5:**
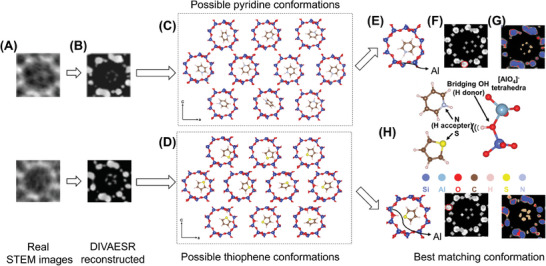
DIVAESR Model‐Assisted Single Molecule Conformation Matching and Determination of Al Sites in Zeolites. A) Real iDPC‐STEM images of thiophene and pyridine. B) Reconstructed images C,D) Possible conformations of pyridine and thiophene. E) Determination of the most similar conformation by SSIM scores. F) Labels G) Element type analysis and single molecule localization through pixel value clustering of reconstructed images. H) Schematic diagram of thiophene and pyridine molecules interacting with Brønsted acid sites via hydrogen bonds and van der Waals forces.

In the field of zeolite research, incorporating aluminum (Al) atoms into the zeolite structure is critical for enhancing the functionality of these molecular sieves. The replacement of tetravalent silicon (Si) atoms with trivalent aluminum atoms at the tetrahedral sites (T‐sites) introduces negative charges into the zeolite framework.^[^ ^14^ ^]^ These negative charges are balanced by protons, leading to the formation of Brønsted acid sites. These sites are the active catalytic centers within the molecular sieves, providing zeolites with their superior catalytic and separation properties. Experimental evidence suggests that the combined effect of bifurcated three‐center hydrogen bonds and van der Waals (VDW) interactions is pivotal in stabilizing the near‐horizontal orientation of aromatic small molecules (**Figure** **5**H).^[^ ^15^ ^]^

However, directly distinguishing Si from Al atoms to identify intrinsic acid sites in experimental images (Figure 5A) is a significant challenge due to their very close relative atomic number. Our DIVAESR model becomes instrumental here, providing higher contrast in reconstructed iDPC‐STEM images (Figure 5B). This enhancement allows for atomic‐level differentiation, which facilitates a clustering analysis based on the distribution of pixel values (contrast) (Figure 5G) and enables the rapid identification of coordinates for framework Si and O atoms, as well as the coordinates of small molecules within the pores. This is essential for understanding the single‐molecular diffusion mechanisms, as the positions of these atoms and their surrounding environments are directly linked to the efficiency and selectivity of the molecular transport process.

Furthermore, by varying the locations of Al atoms at the ten equivalent T‐sites, we can derive all optimized conformations for the framework and encapsulated single molecules (pyridine or thiophene) through DFT calculations (Figure 5C,D). By performing image similarity analysis between these calculated low‐energy conformations and experimental images, and evaluating the DFT results with the SSIM (Figure 5F and Figures S5, S6 and S7, Supporting Information), we were able to quickly pinpoint the locations of Al T‐sites within the zeolite structure (Figure 5E).

## 3 Conclusion

In summary, our research has developed an effective deep learning framework in the field of scanning transmission electron microscopy, particularly achieving significant advancements in the precise detection and analysis of single molecules in iDPC‐STEM images across various electron doses. Our work not only continues the trend of enhancing image quality and noise reduction but also significantly diverges from previous studies focused on images with clear structural frameworks. We have pioneered in precisely characterizing single molecules within the channels of zeolites. The DIVAESR model notably enhances image quality and noise reduction, laying a solid foundation for precise molecular detection and analysis. We employed PSNR, VIF, and SSIM as key metrics to evaluate the performance of the DIVAESR model from three perspectives: image reconstruction quality, visual information fidelity, and structural similarity. The test results confirmed that the model not only preserves the original image information but also significantly enhances image quality.

Furthermore, in the detection task based on reconstructed images and the Faster‐RCNN model, our framework demonstrated exceptional accuracy in identifying molecules in both simulated and actual iDPC‐STEM images. Notably, through clustering analysis of the reconstructed experimental images and DFT calculations, we were able to effectively pinpoint the locations of Al T‐sites within the zeolite frameworks. This research showcases the effective use of machine learning in addressing the complex challenges of characterizing materials that are sensitive to electron beams within the field of electron microscopy. It not only advances the exploration of catalytic sites at the molecular level within zeolites and the analysis of intermolecular interactions but also opens new pathways for real‐time monitoring of dynamic changes in intermediates during chemical reactions. This contributes to a deeper understanding of chemical reaction mechanisms and the development of porous materials research. Despite these advancements, the generalizability of our model remains limited due to data constraints. In the future, with access to a more diverse range of data and experimental images featuring various conformations, this research framework will possess greater generalizability and applicability, thereby playing a significant role in numerous fields.

## 4 Extended Table

**Table 1:** Comparison of Performance with VAE Models and Variants.

Model	Batch size	Learning rate	Epoch	MSE Error
VAE	64	0.001	120	96.84
SR	64	0.001	120	NA
VAESR	64	0.001	120	59.49
**DIVAESR (Ours)**	**64**	**0.001**	**120**	**51.68**

**Table 2:** Determine the best parameter (λ) to balance Los*s_DIVAE_* and Loss_SR_ (NA indicates that the Mean Squared Error (MSE) is excessively high).

*L_DIVAESR_* = *L_DIVAE_* + λ · *L_SR_*	
λ	MSE Error
**0.1**	**51.91**
0.2	55.99
0.5	71.75
0.7	123.37
1	2298.17
Only SR Loss	NA
Only DIVAE Loss	3900.82

## Conflict of Interest

The authors declare no conflict of interest.

## Supporting information



Supporting Information

## Data Availability

The data that support the findings of this study are available in the supplementary material of this article. The source code to implement the machine learning tasks within this study are available from GitHub (https://github.com/yyt-2378/phase_structure_recognition).
