# Supplementary material for: Deep Learning‐Enabled STEM Imaging for Precise Single‐Molecule Identification in Zeolite Structures
Source: Adv Sci (Weinh). 2024 Dec 20;12(6):2408629. doi: 10.1002/advs.202408629 (PMC11809325; doi:10.1002/advs.202408629)
Supplement: Supplementary file 1 — Supporting Information [file ADVS-12-2408629-s001.docx]

Supplementary Materials for

***Deep learning-enabled STEM imaging for precise single-molecule identification in zeolite structures***

Yaotian Yang^1†^, Hao Xiong^1,2†^, Zirong Wu^1^, Zhiyao Luo^1^, Xiao Chen^1,2*^, Xiaonan Wang^1,2*^,

Fei Wei^1,2^

Correspondence to: chenx123@tsinghua.edu.cn (X.C.);

wangxiaonan@tsinghua.edu.cn (X.W.);

†These authors contributed equally to this work.

*Corresponding author. Email: chenx123@tsinghua.edu.cn (X.C.); wangxiaonan@tsinghua.edu.cn (X.W.)

**The supporting information shown below includes the following:**

Materials and Methods

**1.**Experiment details in page 2-5;

**2.**Details of Reconstructing models in page 5-8;

**3.**Details of Recognition model in page 8-9;

**4.**The program flow and principles of the CIF2Label program in page 9-10;

**5.**Detail of Single molecular information acquisition in page 10;

**6.**Explanation of SSIM Score Discrepancies between Thiophene and Pyridine in page 10.

Figs. S1 to S14 (Page 11-30)

Tables S1 (Page 31)

Materials and Methods

Section 1: Experiment details

abTEM generates simulated iDPC-STEM images

The iDPC-STEM images were simulated using open-source abTEM software with Kirkland’s parametrization of atomic potentials, implementing the multislice method^[1]^. The simulation parameters were set to match experimental values, including accelerated voltage (300 kV), convergence angle (15 mrad), collection angle (4~22 mrad), and pixel size (0.2514 × 0.2514 Å^2^). The aberrations were set to zero for simplicity.

Thermal diffuse scattering (TDS) and shot noise were considered in the simulation process. Twenty frozen phonon configurations were averaged to account for TDS. The standard deviations for the random thermal motions of Si and O were 0.075 Å and 0.1 Å, respectively. Poisson noise is added to convergent beam electron diffraction (CBED) patterns to simulate the influence of different shot noise levels (ranging from 500 to 10^6^ e^-^/Å^2^). Simulated iDPC images were calculated for different doses based on the configured virtual 4-quadrant detector, which is further illustrated in Fig. S8 in the supplementary material. Finally, simulated iDPC images were convolved with a Gaussian in order to include the effect of the finite source size (~80 pm).

Considering the synthesized ZSM-5 zeolite samples with quasi-two-dimensional thin regions (~4 nm), single pyridine or thiophene molecule are placed into two-unit-cell-thick ZSM-5 atomic structures with different Al siting. DFT calculations were utilized to determine optimal adsorption conformation which are used to generate the corresponding iDPC images.

Experimental single-molecular iDPC-STEM imaging

The iDPC-STEM experiments were performed using a Cs-corrected scanning transmission electron microscope (FEI Titan Cubed Themis G2 300) operated at 300 kV equipped with a 4-quadrant detector. The convergence semi-angle was 15 mrad, the beam current was lower than 0.5 pA, the collection angle was 4–22 mrad, and the dwell time of probe scanning was 32~64 μs.

Single pyridine and thiophene molecules were confined in the straight channels of ZSM-5 zeolites with ultrathin areas (4~6 nm) as previously reported^[1a, 2]^. Sheet-like ZSM-5 single crystals were dispersed in the pyridine and thiophene pure liquid under ultrasound for 1~2 hours. Then the samples were transferred to microgrids and heated to 130 °C for 2 h to remove physically adsorbed molecules, and only remain those strongly interacting with acid sites.

First-principles calculations

Periodic DFT calculations were carried out using the generalized gradient approximation (GGA) as formulated by Perdew-Burke-Ernzerhof (PBE), and implemented in the Vienna ab initio simulation package (VASP 5.4.5)^[3]^. The valence electronic states were expanded using plane waves, with the core-valence interaction described via the projector augmented wave (PAW) method^[4]^. A kinetic energy cutoff was set at 450 eV. The Γ k-point was exclusively used for sampling the Brillouin zone. The calculations adhered to force and energy convergence criteria of 0.01 eV·Å^−1^ and 1.0 × 10^−5^ eV, respectively, for all structural optimizations.

A periodic siliceous MFI structure (Si_96_O_192_) with lattice dimensions of 20.02 × 19.9 × 13.38 Å^3^ served as the base model. The unit cell of the ZSM-5 was built by replacing one T-site Si atom with an Al atom. The ZSM-5 unit cell was modeled by substituting one T-site Si atom with an Al atom, introducing a charge-balancing proton to the nearest oxygen atom adjacent to Al, forming Brønsted acid sites (O–Si–O(H)–Al–O). Throughout the calculations, all atoms were fully relaxed while keeping the lattice constants fixed.

The DFT-D3 scheme was employed to account for the long-range van der Waals (vdW) dispersion interactions between guest molecules (pyridine and thiophene) and the zeolite framework, ensuring accurate modeling of molecular interactions and stabilization within the ZSM-5 framework^[5]^. Each configuration, as depicted in [Fig. 5C and D](#Fig5), was optimized to have the lowest interaction energy for each acid site position.

Data augmentation

It plays a pivotal role in enhancing the diversity and robustness for training machine learning models. We implement a series of augmentation techniques to simulate different imaging conditions and challenges that might be encountered in real STEM experiments including:

**Brightness variation** is crucial for adapting to images with different levels of brightness, which is common in STEM imaging. It involves adjusting the brightness of images to simulate the instability of electron beam currents.

**Geometric Transformations** are utilized to account for different orientations and positions of molecules in iDPC-STEM images. Such augmentation enables images to be rotated by random angles, and/or be mirrored with a fixed probability. These modifications are crucial for training models to recognize molecular structures regardless of their spatial orientation.

**Cropping and Zooming** techniques are utilized to broaden the model's exposure to different image scales and focal areas. This technique involves selectively focusing on either the center or various sections of the image to capture diverse perspectives and details. Additionally, we utilized a method for adjusting the scale of the image, which involves altering the size of the image and its corresponding labels, enabling the model to handle images with different levels of magnification. These strategies collectively provide the model with a rich training dataset capable of adapting to various imaging conditions.

Incorporating these data augmentation techniques ensures a comprehensive and varied training dataset. Our initial dataset consisted of 92 images with dimensions of 271x406. After data augmentation, the dataset expanded to approximately 11,000 images of size 128x128, along with their corresponding labels. We have used this augmented dataset to train our model. This diversity is key to developing a robust model capable of accurately identifying molecular structures across a wide range of imaging conditions typically encountered in STEM analysis.

DIVAESR Model Training

The DIVAESR model encompasses two sub-models: DIVAE for denoising and EDSR for super-resolution. We initiate with denoising, where the dataset of noisy images $X$ consists of images $x_{i}\in X$, and their ideal noise-free pairs $x_{i}^{'}\in X^{'}.$We assume the noise can be eliminated by a denoising neural network $f:X\to X'$ parameterized by a set of weights $\theta$, producing denoised images  $\hat{x}=f\left( x;\theta\right)$

The objective of the denoise model is then given by

$$\begin{aligned} \underset{\theta}{\mathrm{argmin}} \mathbb{E}_{x_{i}\sim X,{x^{'}}_{i}\sim X^{'}}\left\| f\left( x_{i};\theta\right)-x_{i}^{'} \right\|_{2}\#(S1) \end{aligned}$$

Subsequently, super-resolution enhances the denoised images $\mathbf{X}_{\text{denoise}}$ to a high-resolution dataset $X_{\text{HR}}$. The goal is to map $\hat{x}$to its high-resolution counterpart $\hat{x}_{\text{HR}}$ in $X_{\text{HR}}$ using a super-resolution network$g(\cdot;\phi)$, reconstructing high-resolution images $\hat{x}_{\text{HR}}$.

The DIVAE serves as the initial phase, primarily focused on noise reduction within iDPC-STEM images. Its pivotal role is to isolate essential molecular structures from the prevalent background noise. The DIVAE architecture comprises an encoder and a decoder. The encoder, denoted as $E_{\text{DIVAE}}$, transforms a noisy image $x_{i}$ into a latent space representation $z_{i}$, thereby compressing the data into a lower-dimensional space. This encoding process effectively captures vital features and filters out noise. Subsequently, the decoder, denoted as $D_{\text{DIVAE}}$, reconstructs a denoised image $\hat{x_{i}}$, serving as an approximation of the clean image ${x'}_{i}$. The effectiveness of DIVAE stems from its uniquely designed loss function, which specifically targets the noise components within images. This function incorporates a Mean Squared Error term $L_{MSE}$ or pixel-wise accuracy, as well as a Kullback-Leibler divergence $D_{KL}$ term that ensures the encoded latent variables conform to a standard normal distribution, thus guaranteeing model stability and reducing the risk of overfitting.

After DIVAE reduces noise, the Enhanced Deep Super-Resolution (EDSR) module plays a pivotal role in improving image resolution. EDSR is designed to upscale the resolution of $X_{\text{denoise}}$ to $X_{\text{HR}}$, capturing spatial hierarchies of features. The model's structure, based on a convolutional neural network (CNN), omits batch normalization layers to enhance performance and reduce memory usage. The loss function for EDSR calculates the absolute differences between the super-resolved images and the high-resolution targets, preferring the L1 loss function for its ability to produce sharper images.

**DIVAESR Loss Function:** Equation 2 is the total loss formula of the DIVAESR model, Equation 3 is the loss function for the DIVAE component, and Equation 4 is the loss function for the component.

$$\begin{aligned} L_{DIVAESR}=L_{DIVAE}+\lambda\cdot L_{SR}\#(S2) \end{aligned}$$

$$\begin{aligned} L_{DIVAE}\left( \theta,\phi;x,{x^{'}}_{i} \right)=D_{KL} (q_{\phi}(z|x)\left\| p\left( z \right) \right.)+\mathbb{E}_{x_{i}\sim X,{x^{'}}_{i}\sim X^{'}}\left\| \left( x_{i}-f\left( x_{i};\theta\right) \right)-\left( x_{i}-x_{i}^{'} \right) \right\|_{2}\#(S3) \end{aligned}$$

$$\begin{aligned} L_{SR}(\hat{X}_{\text{HR}},X_{\text{HR}})=\mathbb{E}_{x_{HR}\sim X_{HR}, \hat{x}_{HR}\sim\hat{X}_{HR}}\left\| \hat{X}_{\text{HR}}-X_{\text{HR}} \right\|_{1}\#(S4) \end{aligned}$$

Here in the loss function, L_DIVAE_ represents the loss from the Denoising Inference Variational Autoencoder, while L_SR_ corresponds to the loss from the Super-Resolution component. The parameter λ is used to balance the weights between these two loss terms, with the optimal parameter λ presented in Extended Table 2 in the main text.

The DIVAESR framework is a composite of the DIVAE and EDSR modules, creating a seamless two-step image enhancement process. Initially, DIVAE focuses on reducing noise within iDPC-STEM images, thereby isolating crucial molecular details. Subsequently, EDSR takes over to upscale these denoised images to higher resolutions. This complex process involves a series of loss metrics that finely tune the model's performance. The Mean Squared Error (MSE) function is pivotal in assessing pixel-level reconstruction accuracy, while the Kullback-Leibler (KL) divergence ensures that the encoded latent variables closely adhere to their prior distribution, effectively preventing overfitting. At the same time, the Super-Resolution (SR) loss function guides the upscaling of images to match high-quality target values, thus ensuring that the final output images are both high-resolution and significantly denoised. The Determination of Optimal Hyper-parameter Combinations through Grid Search is displayed in Supplementary Table S1.

The sophisticated orchestration of these components culminates in the final output: a high-resolution image with significantly reduced noise and enhanced clarity in detail. Such images are eminently suitable for complex tasks e.g. molecular identification and localization in STEM analysis. Thus, the DIVAESR framework represents a synergistic fusion of denoising and super-resolution techniques, which is crucial for enhancing the quality of iDPC-STEM images and enabling precise molecular analysis. In supplementary Fig. S9, we present the results of the DIVAE denoising and DIVAESR noise reduction enhancement reconstruction effects from the model testing on the validation set during the training process, along with the corresponding labels. Additionally, we have tested the model on the same channel under different electron doses, as shown in Fig. S10.

Faster-RCNN Model Training

Following the image enhancement from Stage One, the Stage Two model training involves the utilization of Faster R-CNN for object detection. This phase requires the model to accurately identify and locate single molecules within the denoised high-resolution images. The training involves feeding the model with images and corresponding bounding box labels that indicate the position of the molecules.

The model is trained using a set of images that have been augmented to represent a variety of electron dose conditions, orientations, and scales. The object detection model employs a region proposal network (RPN) to hypothesize potential molecular locations, followed by a refinement process to accurately delineate the bounding boxes. The network is trained to minimize the localization and classification loss, which collectively assesses the accuracy of the predicted bounding box positions and the correctness of the molecule identification.

Throughout the training process, the model's performance is validated using a separate validation set that is not utilized during the training phase. The robustness and accuracy of the model are further verified through extensive testing on diverse datasets, ensuring its reliability for practical STEM imaging applications. The tests on simulated datasets and real datasets are displayed in Supplementary Fig. S11.

**Section 2: Reconstructing models**

**Preprocess Model frameworks and the training details:** The preprocess model consists of the Denoising Inference Variational Autoencoder (DIVAE) integrated with the Enhanced Deep Super-Resolution (EDSR) model. The details of the model are as follows:

In the DIVAE model, the encoder initially receives an input image with a specified number of channels (in channels) and incrementally extracts features through a series of convolutional layers (based on the architecture of VanillaVAE). The dimensions of these convolutional layers are default set to [32, 64, 128, 256, 512] channels, employing 3×3 convolutional kernels, a stride of 2, and padding of 1, progressively reducing the spatial dimensions of the image while increasing the depth of features. Following each convolutional layer, batch normalization (BatchNorm2d) and LeakyReLU activation functions are applied to enhance the model's nonlinear expression capability and training stability. Subsequently, the output from the final convolutional layer is transformed into two parameters in the latent space: the mean (mu) and log variance (log var), which describe the distribution of the input data within the latent space. The decoder part then reverses the process of the encoder, initially expanding the representation in the latent space to a higher dimension through a fully connected layer, followed by a series of transposed convolutional layers that gradually upsample and reduce the feature dimensions until the spatial dimensions match those of the input image. The final layer adjusts to the target number of channels using a transposed convolutional layer, followed by batch normalization, LeakyReLU activation function, and a 3x3 convolutional layer, culminating with the Tanh activation function to produce the final image data. The training process of the model involves a reparameterization trick that allows for sampling operations within the latent space while making the process differentiable, thereby facilitating optimization through gradient descent. The model's loss function includes a reconstruction loss (based on MSE) and KL divergence, which respectively ensure the accuracy of the reconstructed image and the regularization of the distribution in the latent space. This integrated process, encompassing encoding, reparameterization, decoding, and the computation of the loss function, forms the forward propagation path of the model. Through continuous iterative training, the model learns how to effectively represent and reconstruct input data.

EDSR model represents a convolutional neural network architected for the purpose of image super-resolution, characterized by its profound network depth and efficacious feature learning capabilities. Under the configuration delineated in the EDSR paper, the model meticulously processes and up-samples images through a sequence of convolutions and residual blocks to achieve high-quality image super-resolution reconstruction. Initially, the model employs a pre-processing step, specifically a Mean Shift operation, to normalize input images. This normalization aims to mitigate the average luminance from the images, thereby reducing the complexity of model training. Subsequently, the image data is fed into the model's head module, comprising a convolutional layer that expands the image from its original color channels to 256 feature channels, utilizing a 3×3 convolution kernel for feature extraction. Following the head module is the core of the model, the body module, constituted by 32 residual blocks. Each block contains two convolutional layers, both employing 3x3 kernels, with the input and output feature channels consistently maintained at 256, preserving the depth of the feature maps. Residual blocks incorporate ReLU activation functions to introduce non-linearity and leverage residual learning strategies to expedite the training process and enhance model convergence. The output from the residual blocks is added to the input after being scaled down through residual scaling (res_scale=0.1), bolstering the model's capability to capture intricate details. Post feature extraction and enhancement, the tail module enlarges the spatial dimensions of the feature maps to the target resolution through an up-sampling layer. Depending on the desired magnification factor, the up-sampling process employs PixelShuffle operations to effectively increase the width and height of images while reducing the number of feature channels. For a 2x magnification, for example, each PixelShuffle operation quadratically decreases the number of feature channels while doubling the image dimensions. The final convolutional layer reduces the number of feature channels from 256 back to the number of output color channels, concluding the image reconstruction process. The post-processing step includes a Mean Shift operation, antithetical to the pre-processing step, to denormalize the image data, thereby restoring the original brightness and color distribution of the images. The design of the EDSR model is aimed at achieving precise and natural image super-resolution outcomes through a deep network structure and the effective implementation of feature extraction, residual learning, and up-sampling techniques while maintaining computational efficiency.

DIVAESR model loss function component mathematical derivation:

MSE Loss (Mean Squared Error Loss): The Mean Squared Error (MSE) Loss is a commonly used loss function that quantifies the average squared difference between the predicted values and the ground truth values. In the DIVAESR model, the MSE Loss is likely employed to measure the discrepancy between the reconstructed output and the original input. Mathematically, it is expressed as:

$$\begin{aligned} \text{MSE Loss}=\frac{1}{N}\sum_{i=1}^{N} \left( x_{i}-\hat{x_{i}} \right)^{2}\#(S5) \end{aligned}$$

where $N$ is the number of samples, $x_{i}$ is the ground truth value, and $\hat{x_{i}}$ is the predicted value.

KL Divergence Loss (Kullback-Leibler Divergence Loss): The Kullback-Leibler (KL) Divergence Loss is a measure of the difference between two probability distributions. In the context of the DIVAESR model, the KL Divergence Loss is likely used to regularize the latent space by encouraging the learned distribution to be close to a prior distribution, such as a standard Gaussian distribution. The KL Divergence Loss is defined as:

$$\begin{aligned} \text{KL Divergence Loss}=\sum_{z} q_{\phi}\left( z | x \right)\log\frac{q_{\phi}\left( z | x \right)}{p\left( z \right)} \#(S6) \end{aligned}$$

where $q_{\phi}\left( z | x \right)$ denotes the approximate posterior distribution or the encoder distribution in the variational autoencoder (VAE) framework. It represents the probability distribution of the latent variable $z$ given the input data $z$. The subscript $\phi$ indicates that this distribution is parameterized by $\phi$, which are the parameters of the encoder network. $p\left( z \right)$ represents the prior distribution of the latent variable $z$. In the context of VAEs, it is often chosen to be a simple distribution, such as a standard Gaussian distribution $\mathcal{N}\left( 0, I \right)$, where $I$ is the identity matrix.

L1 Loss (Absolute Error Loss): The L1 Loss, also known as the Absolute Error Loss, calculates the average absolute difference between the predicted values and the ground truth values. In the DIVAESR model, the L1 Loss might be used as an alternative to the MSE Loss for measuring the reconstruction error. The L1 Loss is more robust to outliers compared to the MSE Loss. It is defined as:

$$\begin{aligned} L_{1}Loss=\sum_{i=1}^{N} \left| y_{i}-\hat{y_{i}} \right|\#(S7) \end{aligned}$$

where$n$ is the number of samples, $y_{i}$is the ground truth value, and $\hat{y_{i}}$ is the predicted value.

**SSIM (Structure Similarity Index Measure)**：The SSIM (Structure Similarity Index Measure), as a perceptually motivated structural similarity metric, better aligns with human visual perception. It is derived from the average gray-scale value, standard deviation of grayscale value, and structural similarity of the images. In our work, it is utilized to measure the disparity between the reconstructed image(*x*) and the original image(*y*).

$$\begin{aligned} SSIM\left( x,y \right)=\left( \frac{2\mu_{x}\mu_{y}+C_{1}}{\mu_{x}^{2}+\mu_{y}^{2}+C_{1}} \right)\cdot\left( \frac{2\sigma_{xy}+C_{2}}{\sigma_{x}^{2}+\sigma_{y}^{2}+C_{2}} \right)\#\left( S8 \right) \end{aligned}$$

where:

1. $\mu_{x}$and $\mu_{y}$ represent the average grayscale values of images $x$ and $y$, respectively. They are calculated using the following formula: $\mu_{x}=\frac{1}{N}\sum_{i=1}^{N} x_{i}$ where $N$ is the total number of pixels in the image, and $x_{i}$ is the grayscale value of the $i$-th pixel in image $x$.
2. $\sigma_{x}$ and $\sigma_{y}$ denote the standard deviation of grayscale values in images $x$ and $y$, respectively. They are computed using the following formula: $\sigma_{x}=\left( \frac{1}{N-1}\sum_{i=1}^{N} \left( x_{i}-\mu_{x} \right)^{2} \right)^{\frac{1}{2}}$ where $N$ is the total number of pixels in the image, $x_{i}$ is the grayscale value of the $i$ -th pixel in image $x$, and $\mu_{x}$ is the average grayscale value of image $x$.
3. $\sigma_{xy}$ represents the correlation coefficient between images $x$ and $y$. It is calculated using the following formula: $\sigma_{xy}=\frac{1}{N-1}\sum_{i=1}^{N} \left( x_{i}-\mu_{x} \right)\left( y_{i}-\mu_{y} \right)$ where $N$ is the total number of pixels in the image, $x_{i}$ and $y_{i}$ are the grayscale values of the $i$ -th pixel in images $x$ and $y$, respectively, and $\mu_{x}$ and $\mu_{y}$ are the average grayscale values of images $x$ and $y$, respectively.
4. $C_{1}$ and $C_{2}$ are constants used to stabilize the division with weak denominator. They are defined as: $C_{1}=\left( K_{1}L \right)^{2}$, $C_{2}=\left( K_{2}L \right)^{2}$, $C_{3}=\frac{C_{2}}{2}$, $K_{1}=0.01$, $K_{2}=0.03$, $L=2^{\text{bits per pixel}}-1$ where $L$ is the dynamic range of the pixel values, typically $2^{\text{bits per pixel}}-1$.

The SSIM value ranges between -1 and 1, with 1 indicating perfect structural similarity between the two images. By incorporating the SSIM loss in the objective function, the model is encouraged to generate reconstructed images that are perceptually similar to the original images, capturing both the structural information and the visual quality.

**PSNR (Peak Signal-to-Noise Ratio)**：PSNR (Peak Signal-to-Noise Ratio) is an engineering term that represents the ratio of the maximum possible power of a signal to the power of destructive noise that affects its representation accuracy. It is commonly used to assess the quality of image reconstruction and is simply defined by the mean square error (MSE). The PSNR is calculated using the following formula:

$$\begin{aligned} PSNR=10\cdot\log_{10} \left( \frac{MAX_{I}^{2}}{MSE} \right)\#\left( S9 \right) \end{aligned}$$

where:

1. $MAX_{I}$ represents the maximum possible pixel value of the image. For example, if the image has 8-bit pixels per sample, $MAX_{I}$ would be 255.
2. $MSE$ is the Mean Squared Error between the clean image $I$ and the noisy image $K$. It measures the average squared difference between the pixel values of the two images. The $MSE$ is calculated using the following formula: $MSE=\frac{1}{mn}\sum_{i=0}^{m-1} \sum_{j=0}^{n-1} \left[ I\left( i,j \right)-K\left( i,j \right) \right]^{2}$ where:
   - $m$ and $n$ are the dimensions of the images (height and width, respectively).
   - $I\left( i,j \right)$ represents the pixel value of the clean image $I$ at the position $\left( i,j \right)$.
   - $K\left( i,j \right)$represents the pixel value of the noisy image $K$ at the position $\left( i,j \right)$.

**Section 3: Recognition model**

The Recognition models presented in this study are predicated upon the Faster R-CNN architecture, a state-of-the-art framework that facilitates the expeditious identification of diminutive molecules within the confines of porous structures. The Faster R-CNN model leverages the AlexNet and VGG16 networks as its foundational backbone for the extraction of salient features, exemplifying the seamless integration of pretrained convolutional neural networks (CNNs) into the Faster R-CNN architecture through judicious modifications.

Within the model's hierarchical structure, the backbone network serves as the primary conduit for initial feature extraction, which is subsequently harnessed by a Region Proposal Network (RPN) to generate candidate object regions from the derived feature maps. The RPN employs an AnchorGenerator and MultiScaleRoIAlign with predefined dimensions and aspect ratios to ensure comprehensive feature extraction and object detection. Subsequent to the RPN, the Region of Interest (ROI) Pooling mechanism is applied to the candidate regions, transforming them into a standardized size conducive to further processing. The culmination of this process involves the classification and bounding box regression for each ROI-pooled region. To accommodate the idiosyncrasies of different backbone CNNs, specific adjustments are implemented. In the case of AlexNet, the output channels of the final convolutional layer are set to 256. Conversely, for VGG16, the last max-pooling layer is removed, the initial 10 layers are frozen to preclude updates during training, and the output channels are calibrated to 512 to align with VGG16's feature extraction capabilities. Furthermore, a bespoke BoxHead class supersedes the original box predictor with the classifier section of VGG16, excluding the final layer, to tailor the model more closely to object detection tasks. The nuanced integration and customization of CNNs within the Faster R-CNN architecture underscore its versatility and efficacy in addressing a wide range of object detection challenges, while maintaining a keen focus on the detailed structure and training nuances of utilizing AlexNet and VGG16 as foundational backbones.

**AP (Average Precision)**: The calculation process of AP is as follows: For each category, based on the matching of the predicted boxes and the ground truth boxes, calculate the confidence score of each predicted box and its corresponding Intersection over Union (IOU) value with the true box. Sort the confidence scores in descending order to obtain a score list, where each score corresponds to a predicted box. Starting from the predicted box with the highest score, each predicted box is sequentially taken as a positive sample, and the IOU value of the ground truth box with all preceding predicted boxes is calculated. If the IOU value is greater than a certain threshold, it is considered a negative sample. Based on the calculated precision and recall, a Precision-Recall curve (PR curve) is obtained. By integrating the area under the PR curve, the AP value for that category can be obtained.

**AR (Average recall)**: AR is the maximum recall of a fixed number of detections in each image, calculated based on the proposals in the given image by comparing them with the ground truth boxes.

**Section 4: The program flow and principles of the CIF2Label program**

The CIF2Label program takes a .cif file as input, which contains information about the atomic structure of the sample. Based on Equation S6, the program identifies the information of all atoms in the input file, including their atomic numbers, coordinates, and radii. Using these information, the CIF2Label program determines the contrast value and relative position of each atom in the label image. The contrast values are assigned based on the atomic number, while the relative positions are determined by the coordinates of the atoms. The program also considers the radius of each atom when constructing the label image.

$$\begin{aligned} I^{iCOM}\left( \vec{r_{p}} \right)=\frac{1}{2\pi}\left( \left| \psi_{in}\left( \vec{r} \right) \right|^{2}\star\varphi\left( \vec{r} \right) \right)\left( \vec{r_{p}} \right)\#\left( S10 \right) \end{aligned}$$

Equation S6 shows that the integrated image$I^{iCOM}\left( \vec{r_{p}} \right)$ directly images the phase wavefunction $\varphi\left( \vec{r} \right)$, coordinates of atom$\left( \vec{r_{p}} \right)$ and incident wavefunction$\left| \psi_{in}\left( \vec{r} \right) \right|^{2}$. Meanwhile, phase wavefunction $\varphi\left( \vec{r} \right)$ correlated with atomic number Z Therefore, based on Equation S6, the CIF2Label program identifies the information of all atoms in the input .cif file and determines the contrast value and relative position of each atom in the label image based on the atomic number, coordinates of the atoms and the radius of the atom. As a result, a label image is reconstructed that disregards noise, maintains the relative positions of the atoms, and exhibits contrast values conforming to an ideal distribution. The correlation between the grayscale value of the label image and the atomic number is as shown in Equation S7.

$$\begin{aligned} I^{label}\left( \vec{r_{p}} \right)=\left( 255-BASE_{I}NCREMENT \right)\times\frac{Z}{16}+BASE_{I}NCREMENT\#\left( S11 \right) \end{aligned}$$

where:$I^{label}\left( \vec{r_{p}} \right)$ is the grayscale value of the label image at the position $\vec{r_{p}}$. $Z$ is the atomic number of the atom at position $\vec{r_{p}}$. $BASE_{I}NCREMENT$ is a constant value that determines the contrast scale of the label image.

**Section 5: Single molecular information acquisition**

To expedite the analysis of atomic positions, we employed the DBSCAN clustering algorithm, combined with a custom filtering approach and grayscale threshold segmentation, to autonomously identify and categorize specific atoms (O/Al/Si). Initially, the code loads and converts images to grayscale, subsequently applying clustering analysis on the pixels within predefined grayscale intensity ranges corresponding to distinct atomic species. By setting varied grayscale thresholds, such as 28 to 165 for one species, 160 to 190 for another, and 185 to 255 for the third, the algorithm effectively discriminates between the three atomic types. Furthermore, a bespoke filter scrutinizes the pixel values within each pixel's neighborhood, enhancing the accuracy of the clustering outcome. Ultimately, using matplotlib, scatter plots and contour maps illustrating the distribution of different atoms within the sample are generated and saved, vividly demonstrating the atomic dispersion. This process not only showcases the application of machine learning techniques in the analysis of material science imagery but also illustrates the efficacy of grayscale threshold segmentation and spatial clustering algorithms in recognizing atomic-scale features.

**Section 6: Explanation of SSIM Score Discrepancies between Thiophene and Pyridine**

The differences in SSIM (Structural Similarity Index Measure) scores between thiophene and pyridine observed in our results stem from intrinsic properties related to their atomic structures and corresponding imaging contrasts in STEM. The SSIM metric evaluates the structural similarity between the reconstructed and the original images, where alignment and pixel intensity play crucial roles.

**Atomic Structure Influence**: In STEM imaging, image contrast correlates with atomic numbers due to the interaction of electrons with the sample. Thiophene, with a chemical formula of C_4_H_4_S, includes a sulfur atom (S), whereas pyridine, represented as C_5_H_5_N, contains a nitrogen atom (N). Sulfur's higher atomic number compared to nitrogen leads to stronger electron scattering, thereby producing images with higher pixel intensities for thiophene.

**Impact on SSIM Scores**: Given SSIM's sensitivity to both alignment and intensity variations, the distinct contrast characteristics of thiophene result in higher pixel intensity, simplifying the denoising and enhancement process. Conversely, pyridine's images, with comparatively lower intensity due to the lower atomic number of nitrogen, appear dimmer, which impacts the SSIM evaluation.

**Supplementary Figures:**

**Fig. S1.** Comparison of DIVAE Noise Reconstruction and Original Added Noise Effects.

**
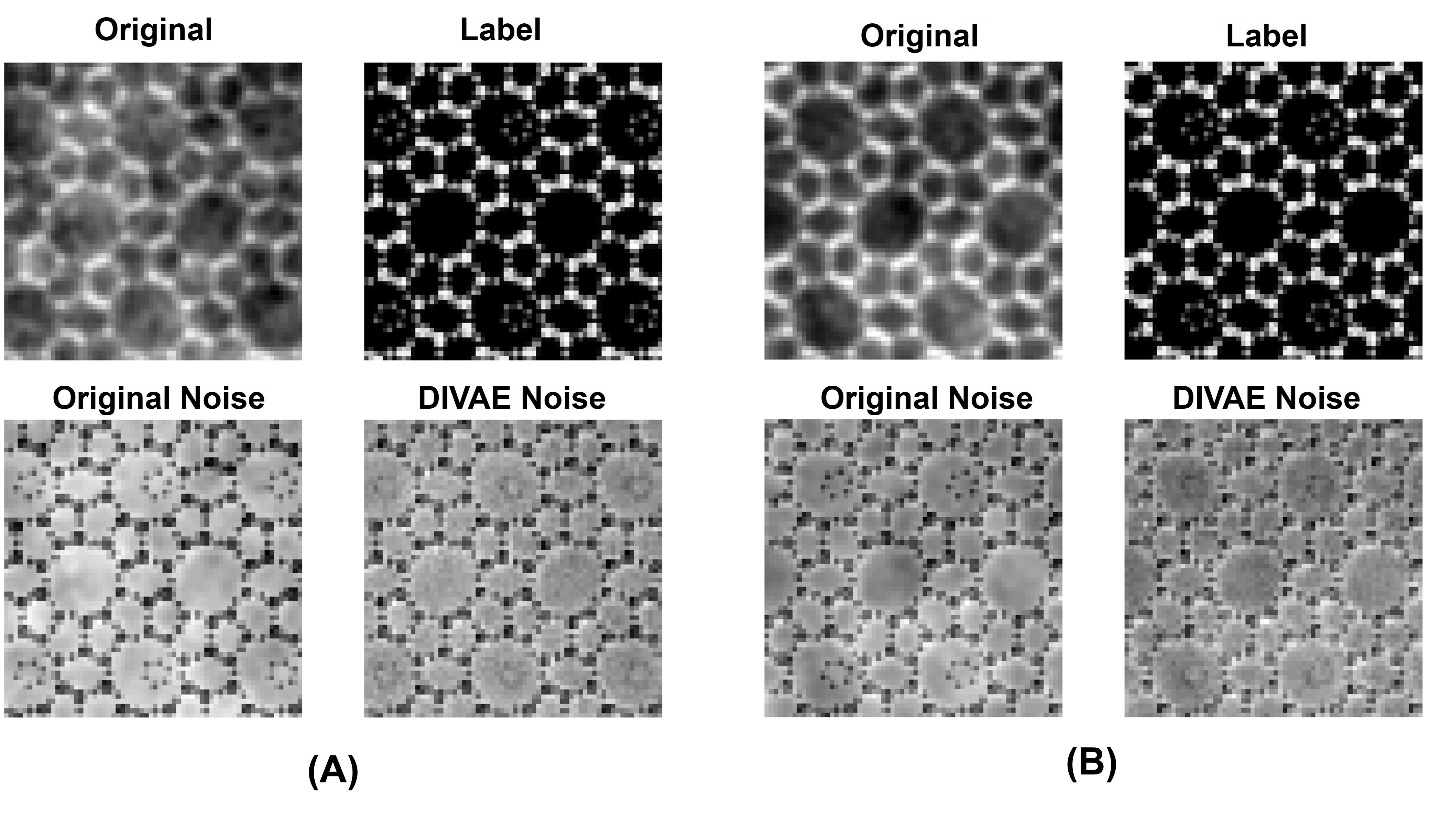

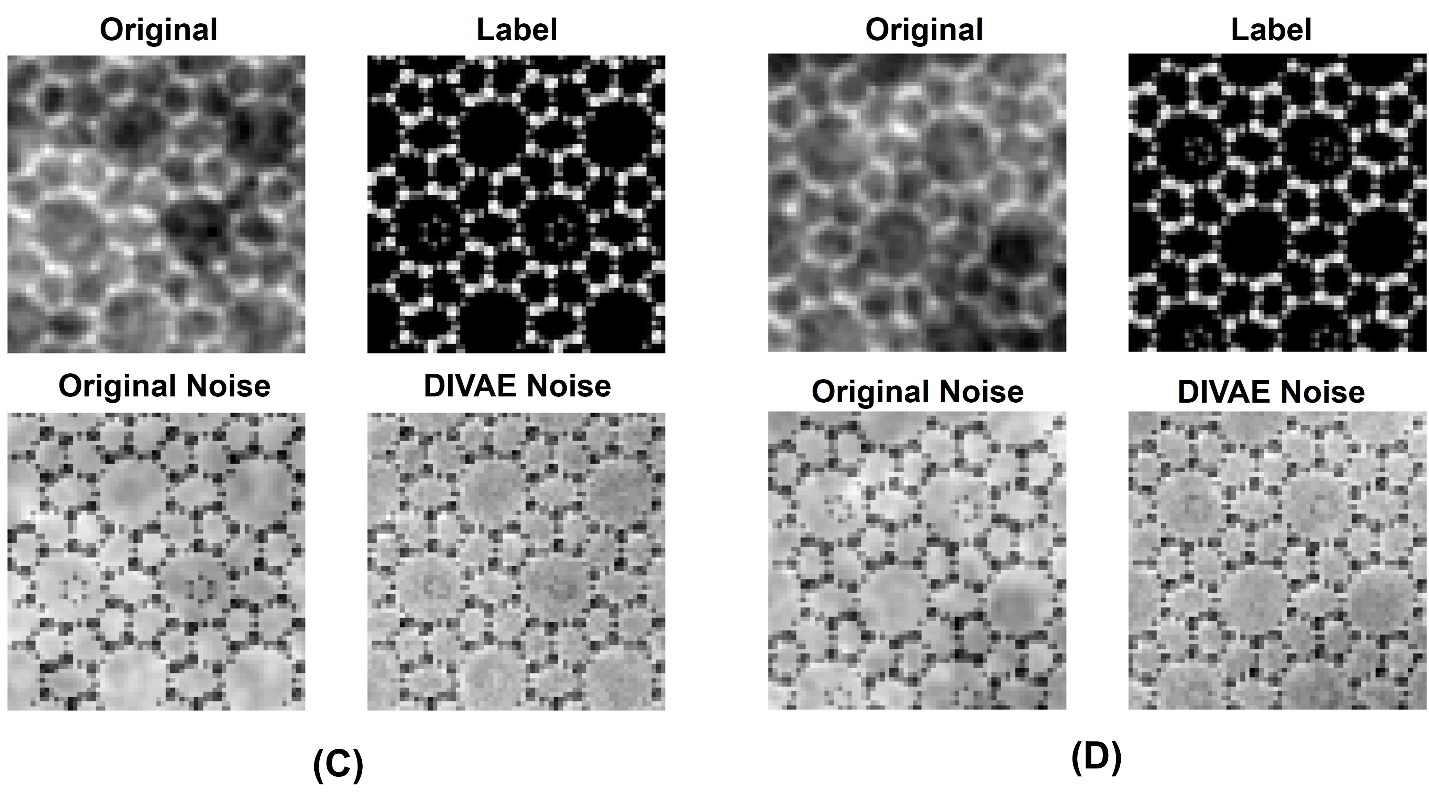
**

**Fig. S2.** Workflow of the Single Molecule Detection Model Framework Using Advanced Image Processing and Machine Learning Techniques and Image Size Variations in the Model.

**
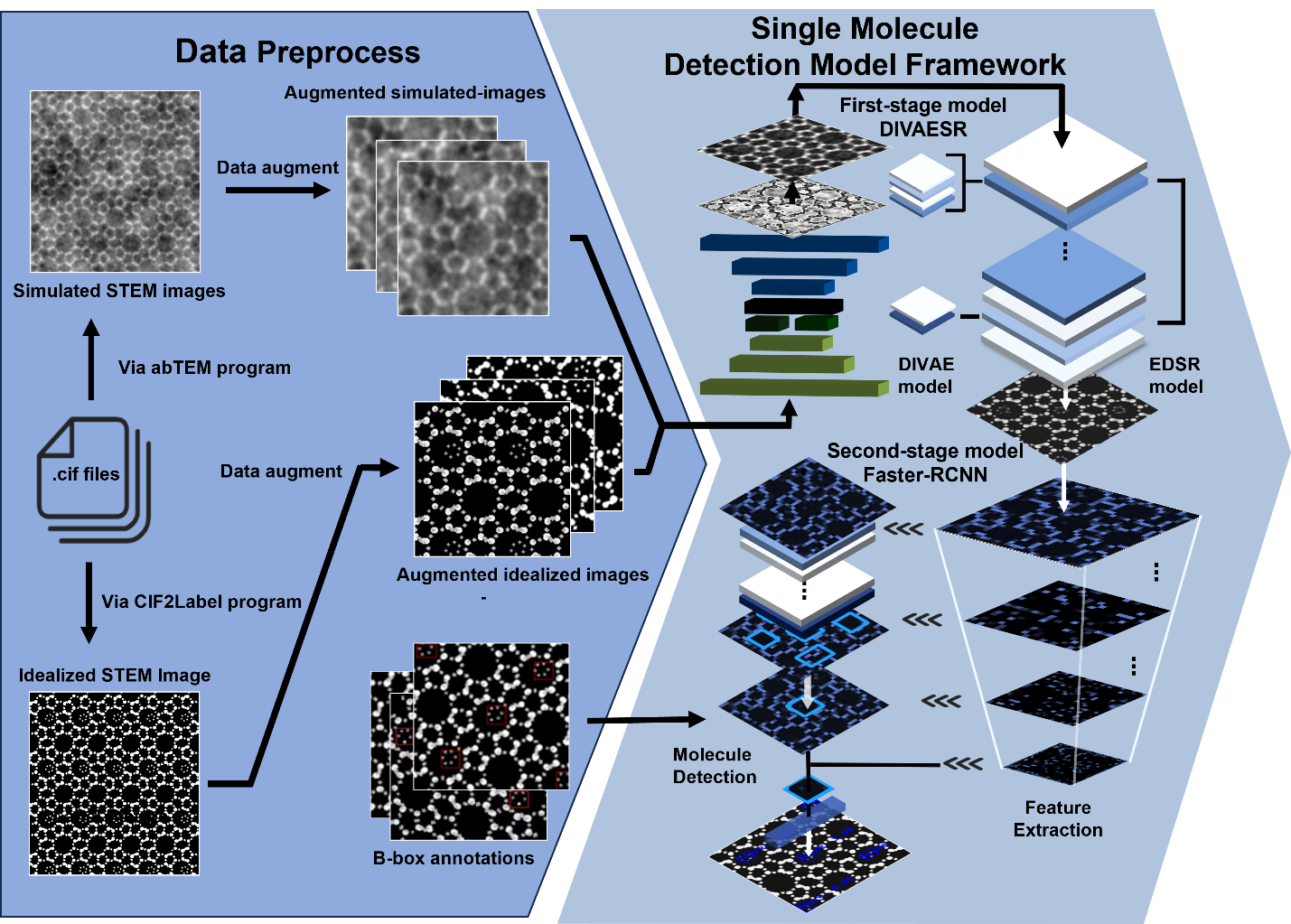
**

**
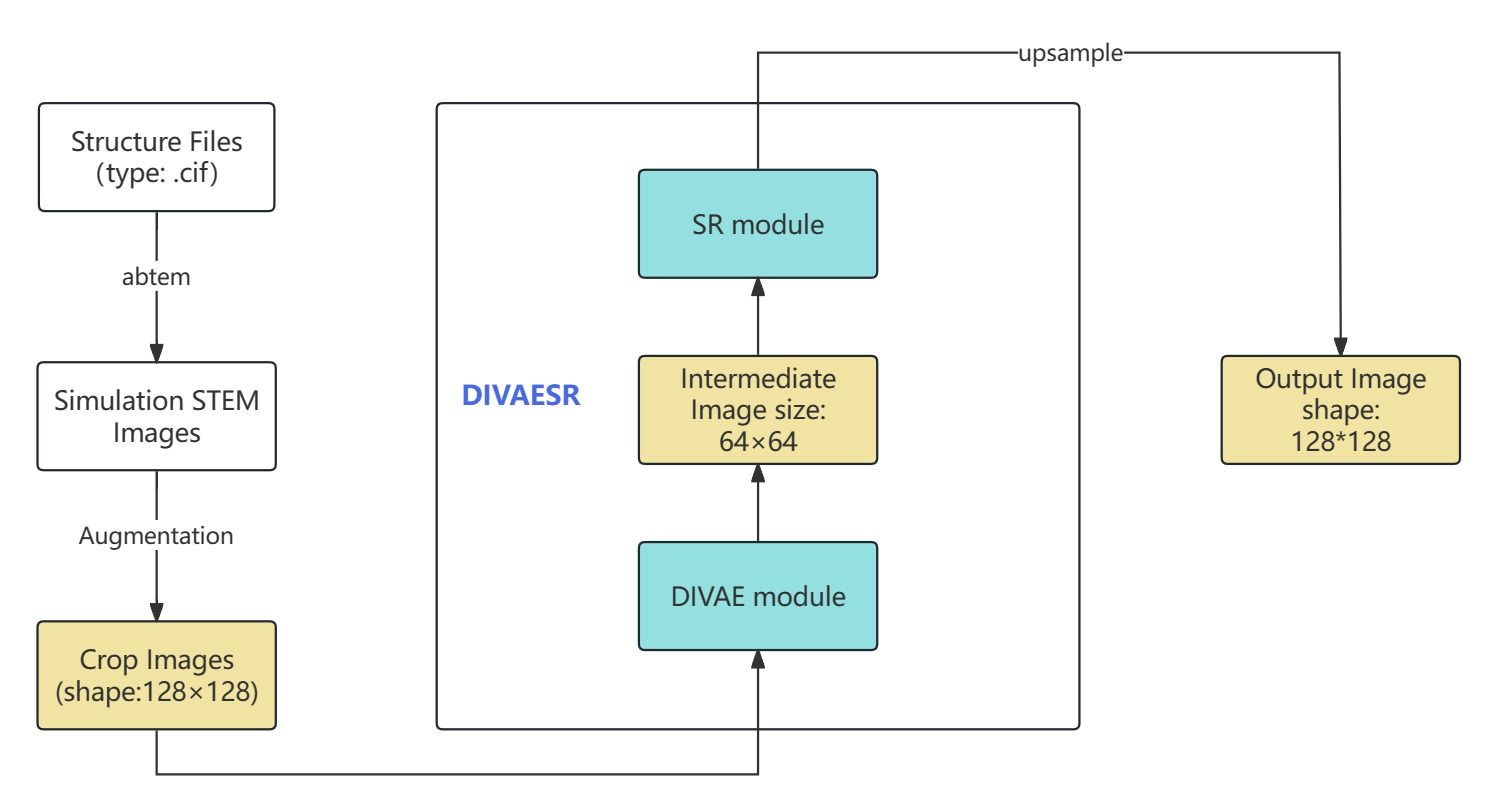
**

**Fig. S3.** Test Set Results Display: Original Image, Label, DIVAE Intermediate and Final DIVAESR Outputs, and Single Molecule Detection Demonstration.

**
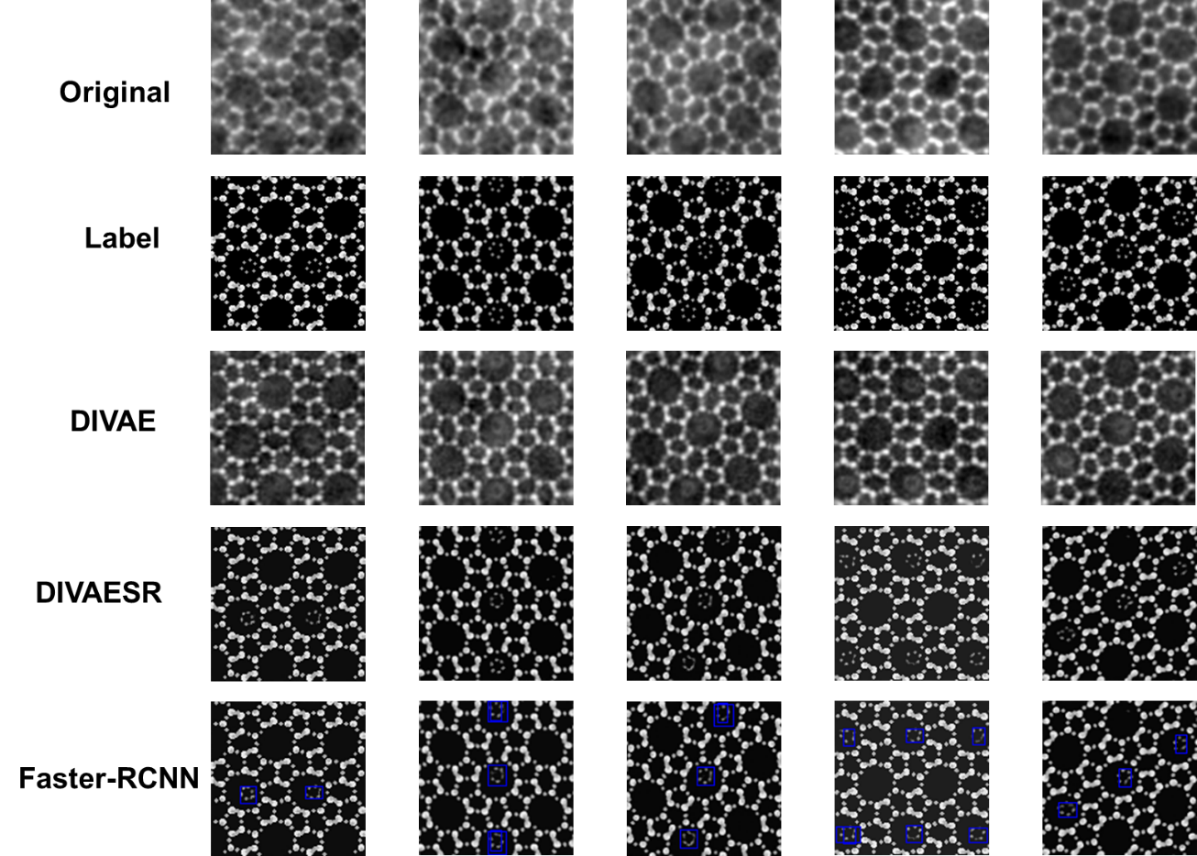
**

**（1）**

**
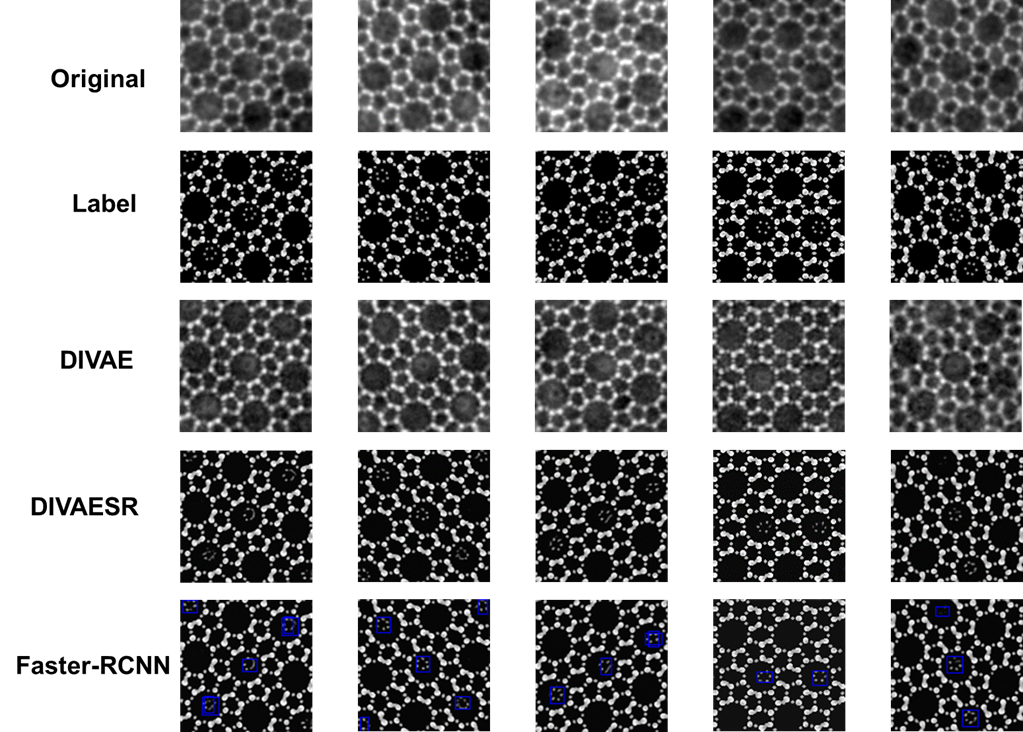
**

**（2）**

**
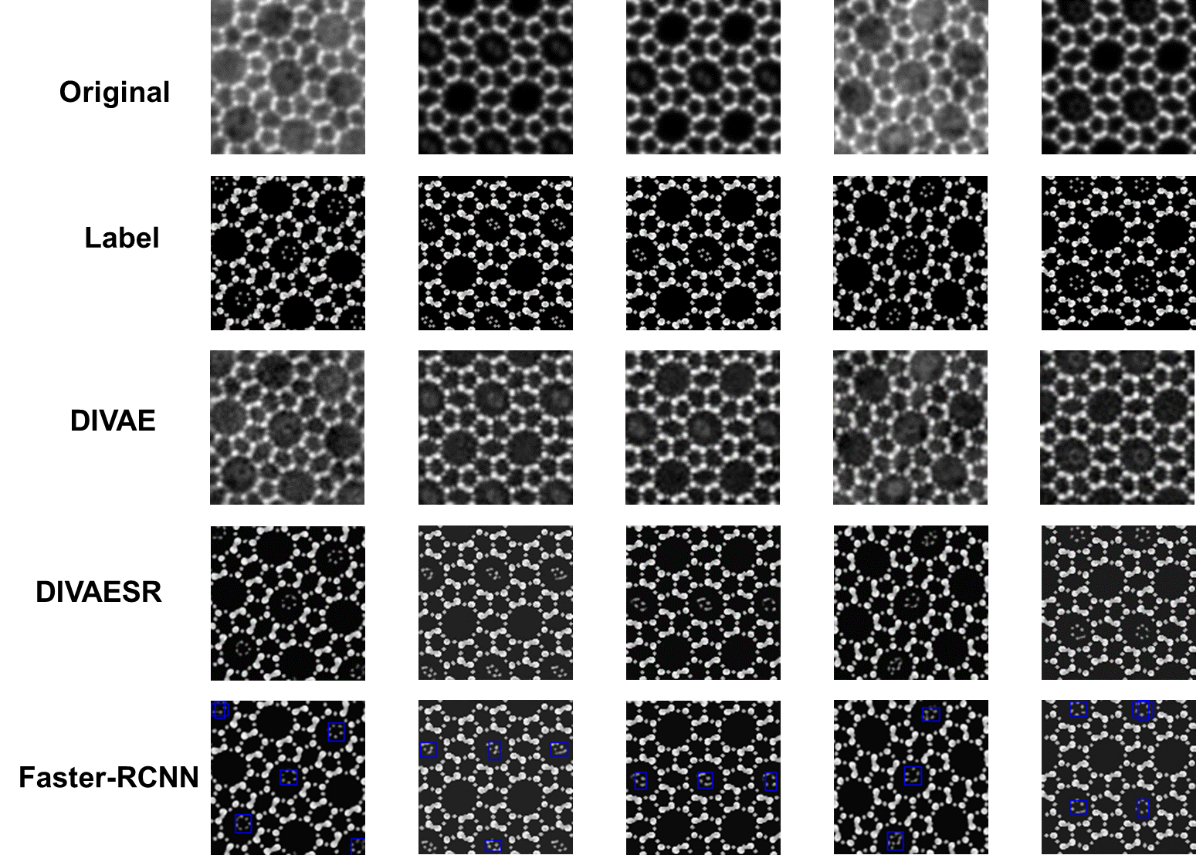
**

**（3）**

**
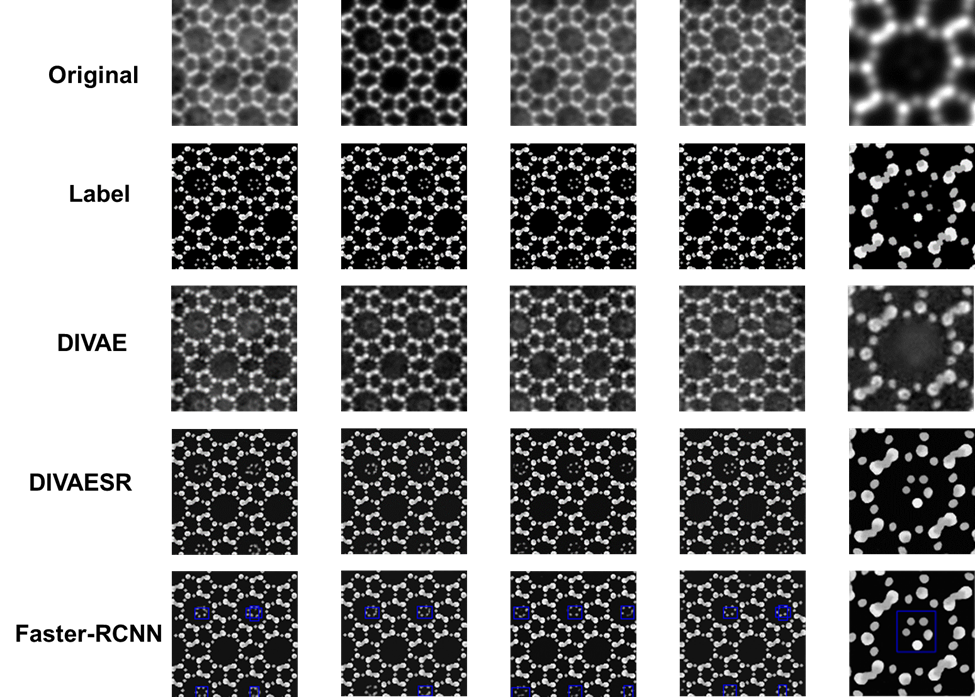
**

**（4）**

**Fig. S4.** Analysis of the test set iDPC-STEM images at low-dose and high-dose, high-resolution and low-resolution.


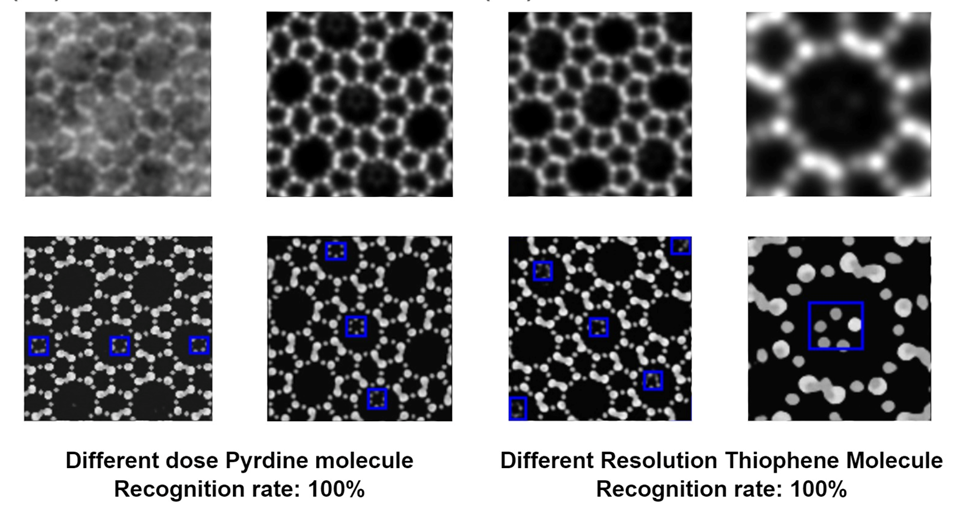


**Fig. S5.** Training dataset conformations.

**
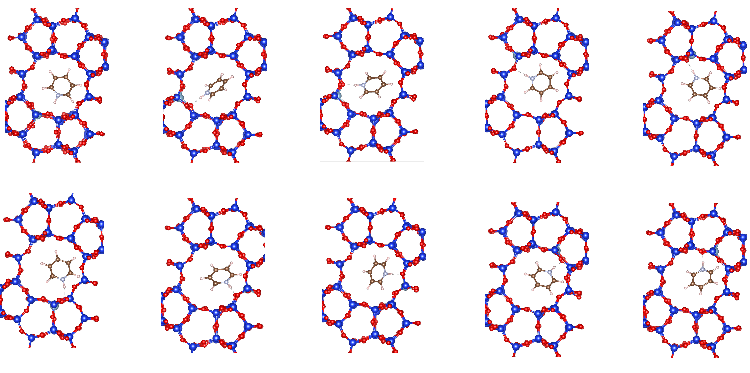
**

**Pyridine training dataset conformations**

**
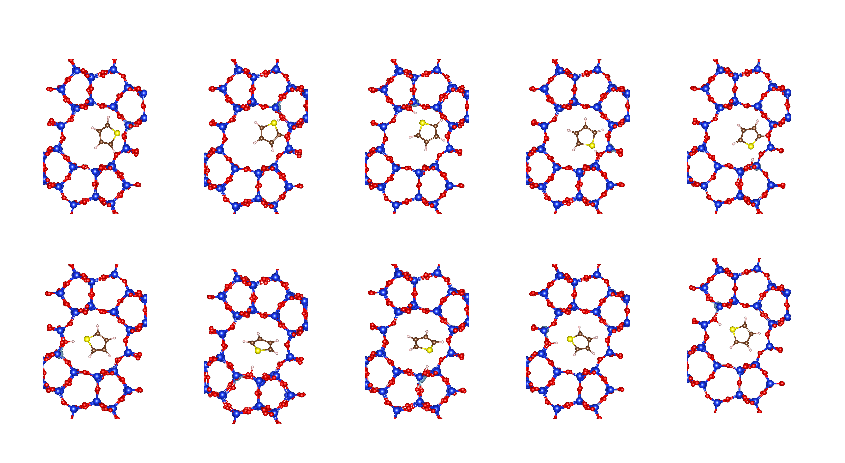
**

**Thiophene training dataset conformations**

**Fig. S6.** SSIM Scores for Different Pyridine Conformation Matching.


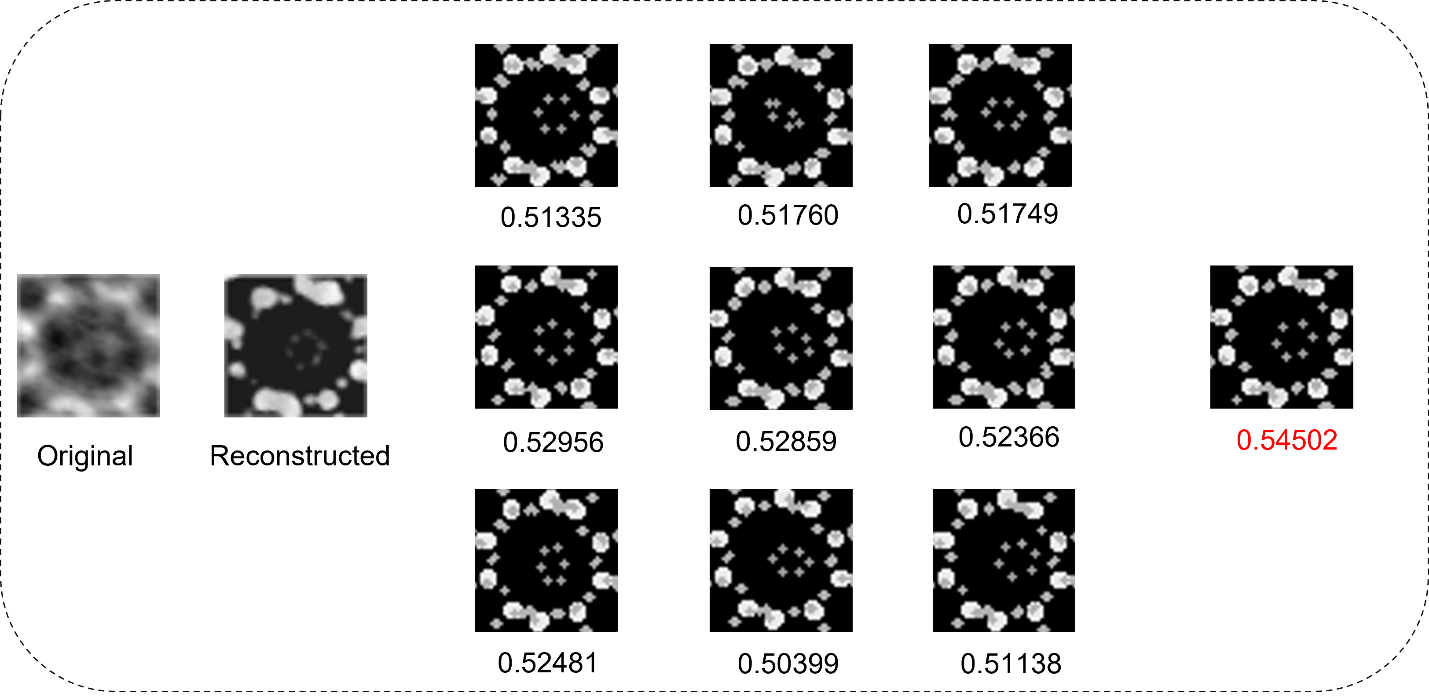


**Fig. S7.** SSIM Scores for Different Thiophene Conformation Matching.

**
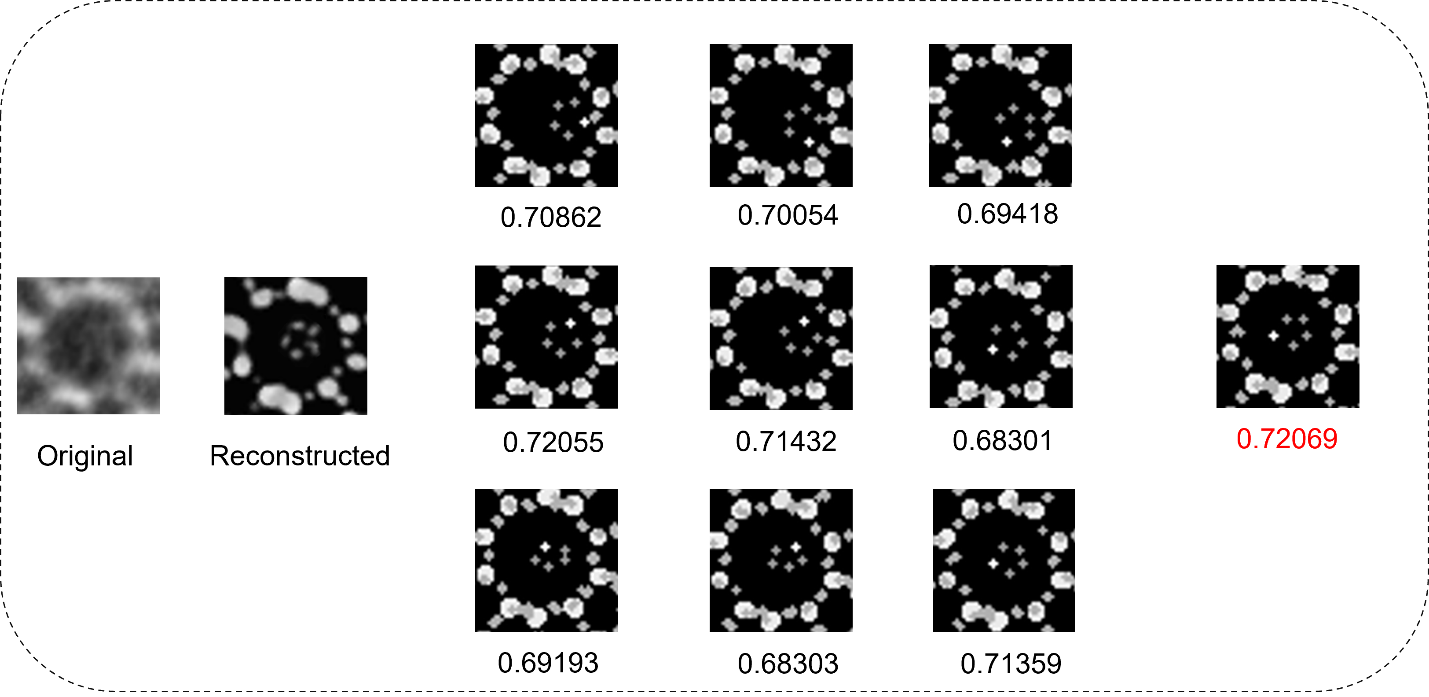
**

**Fig. S8.** Overview of the steps in iDPC-STEM simulation using a 4 quadrant detector.

**
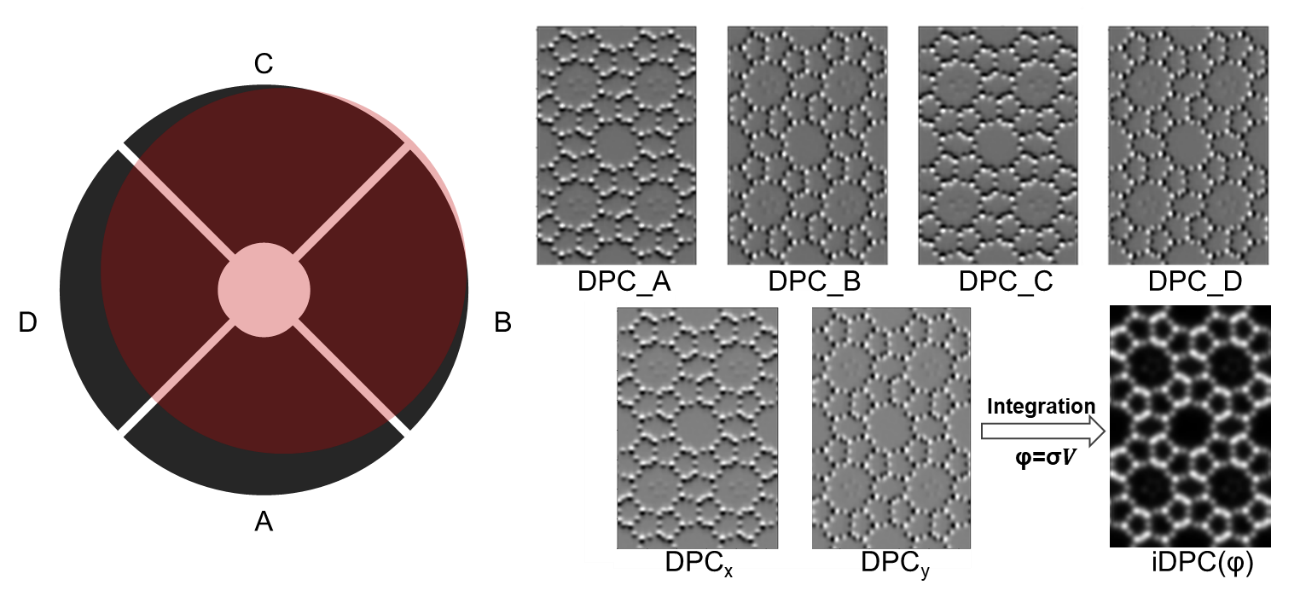
**

**Fig. S9.** The display of reconstruction effects by DIVAESR are as follows: DIVAE reconstruction, SR reconstruction, and sample label training performance images.

**
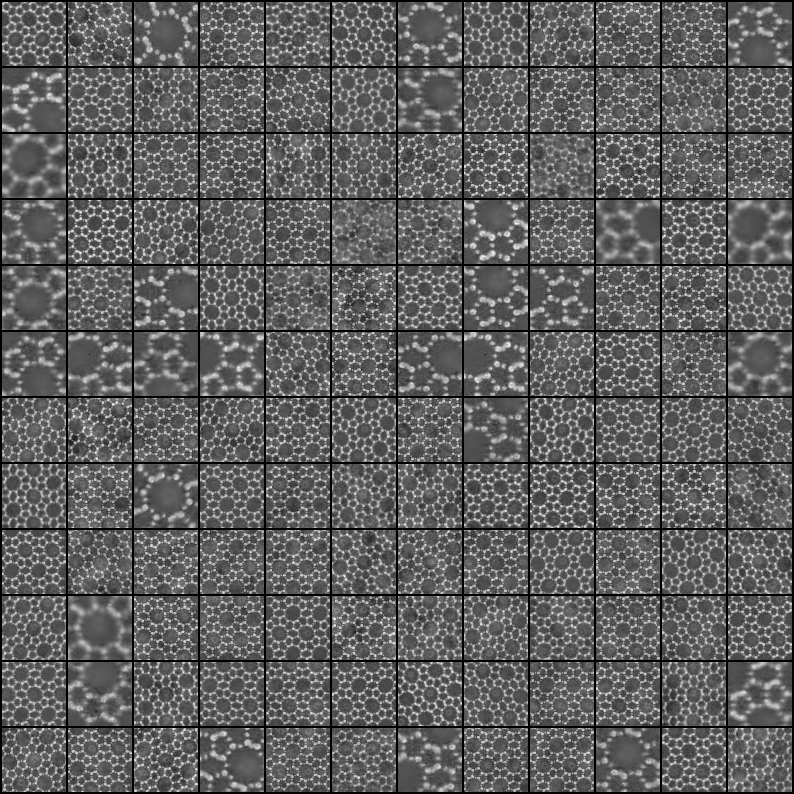
**

**DIVAE Reconstruction (100th Training Performance Display)**

**
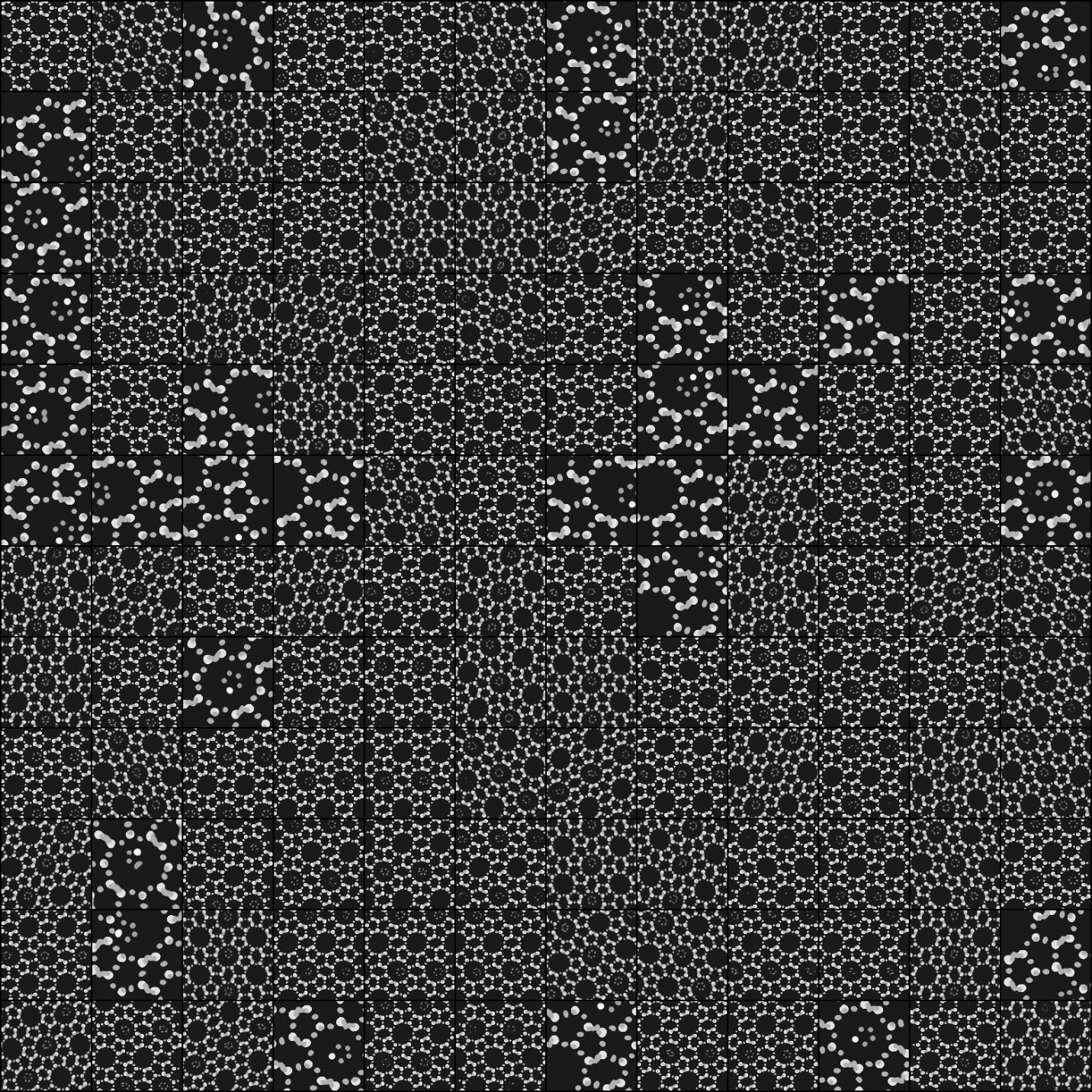
**

**DIVAESR Reconstruction (100th Training Performance Display)**

**
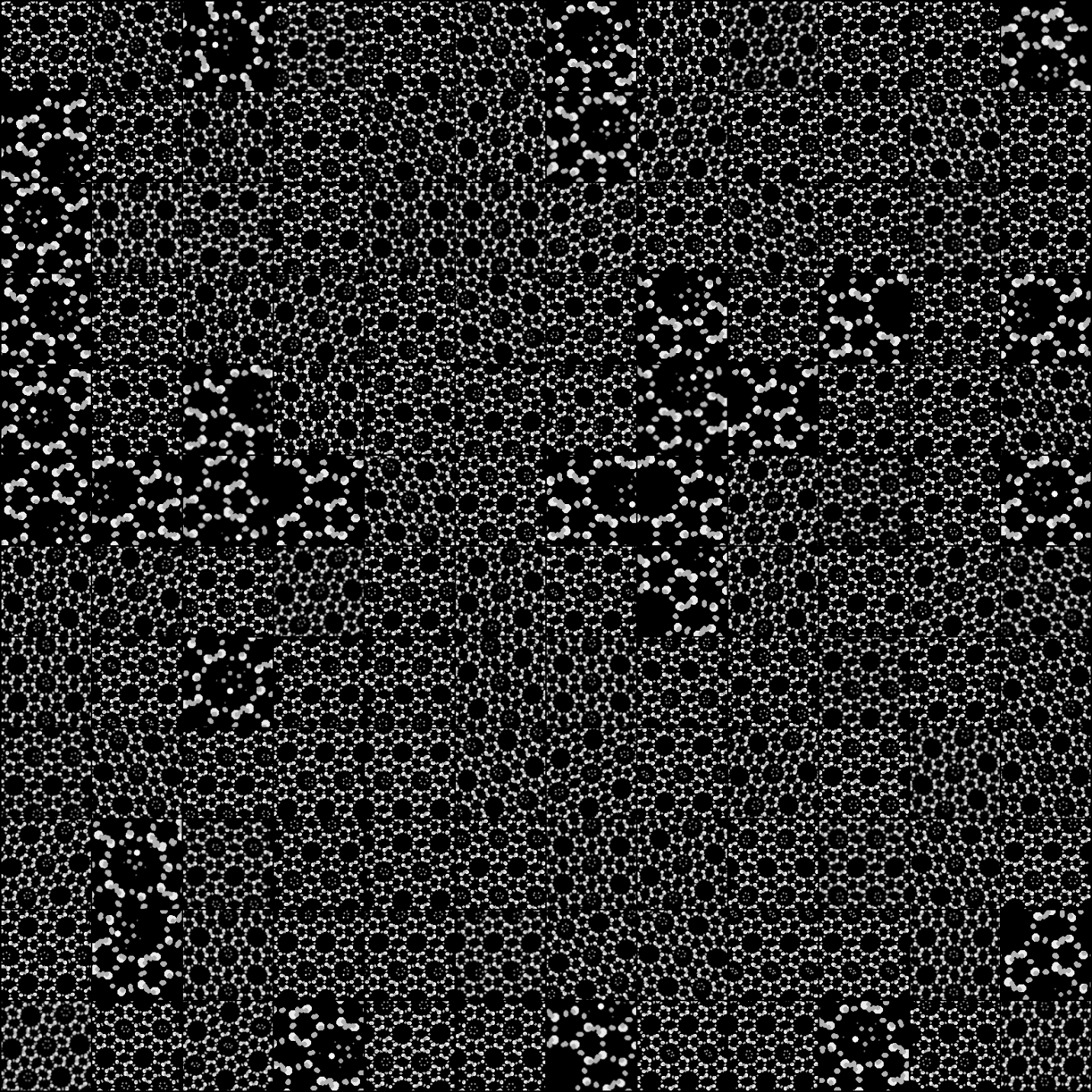
**

**Corresponding Label (100th Training Performance Display)**

**Fig. S10.** Impact of Varying Electron Doses on the Reconstruction of the Same Pore.

**
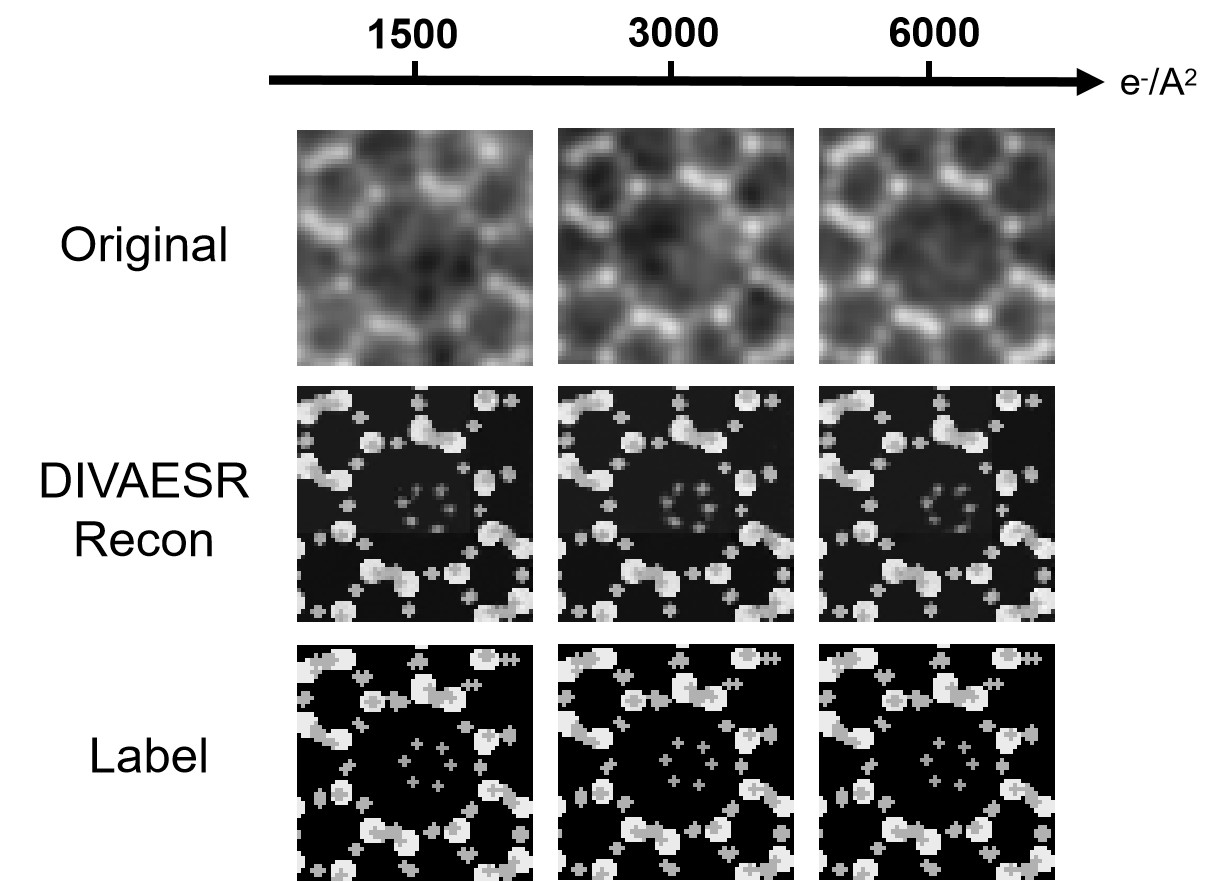
**

**Fig. S11.** Faster R-CNN Large-Scale Visualization: Simulation STEM Image Results and Real STEM Image Results (Pyridine).

| **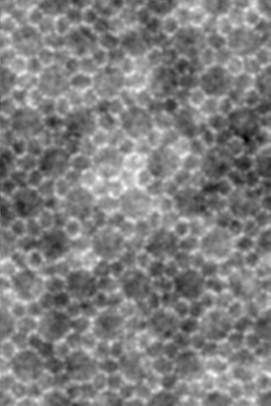** | **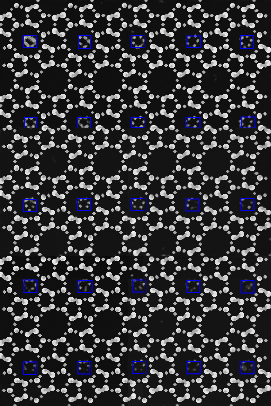** |
| --- | --- |

**Simulation STEM Image Results**

**
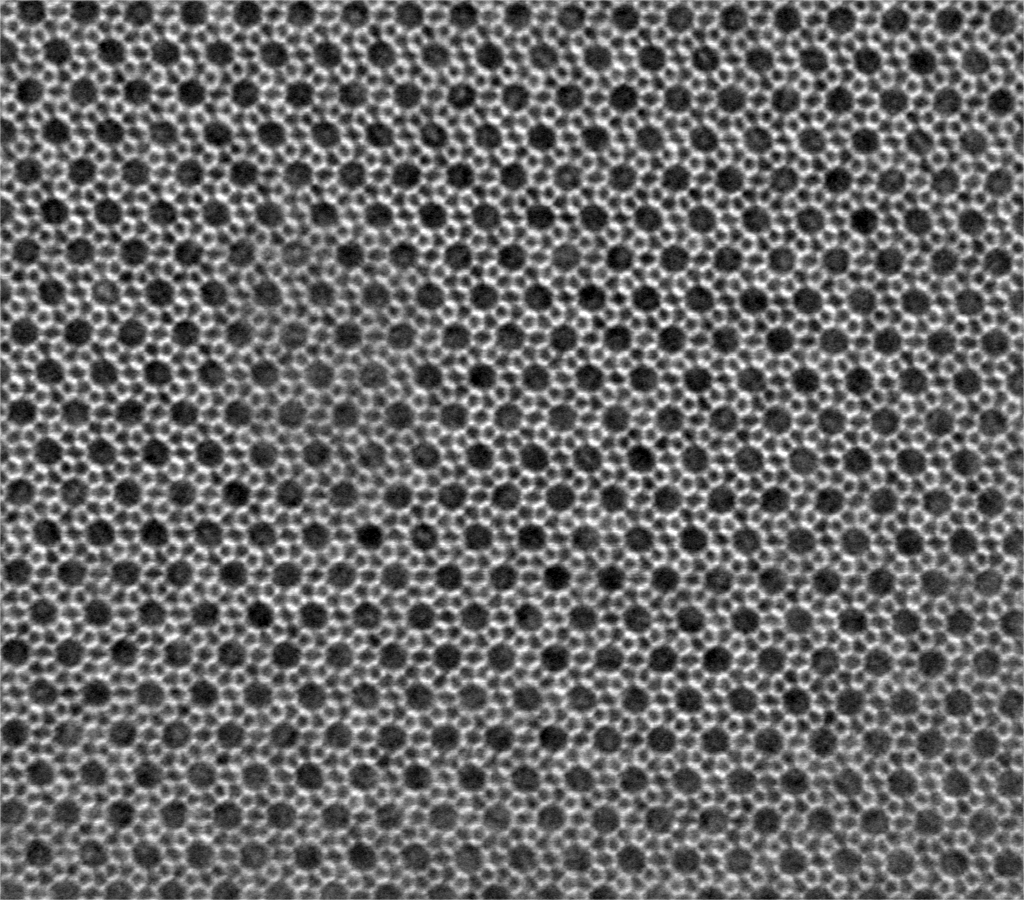
**

**Pyridine real STEM Image**

**
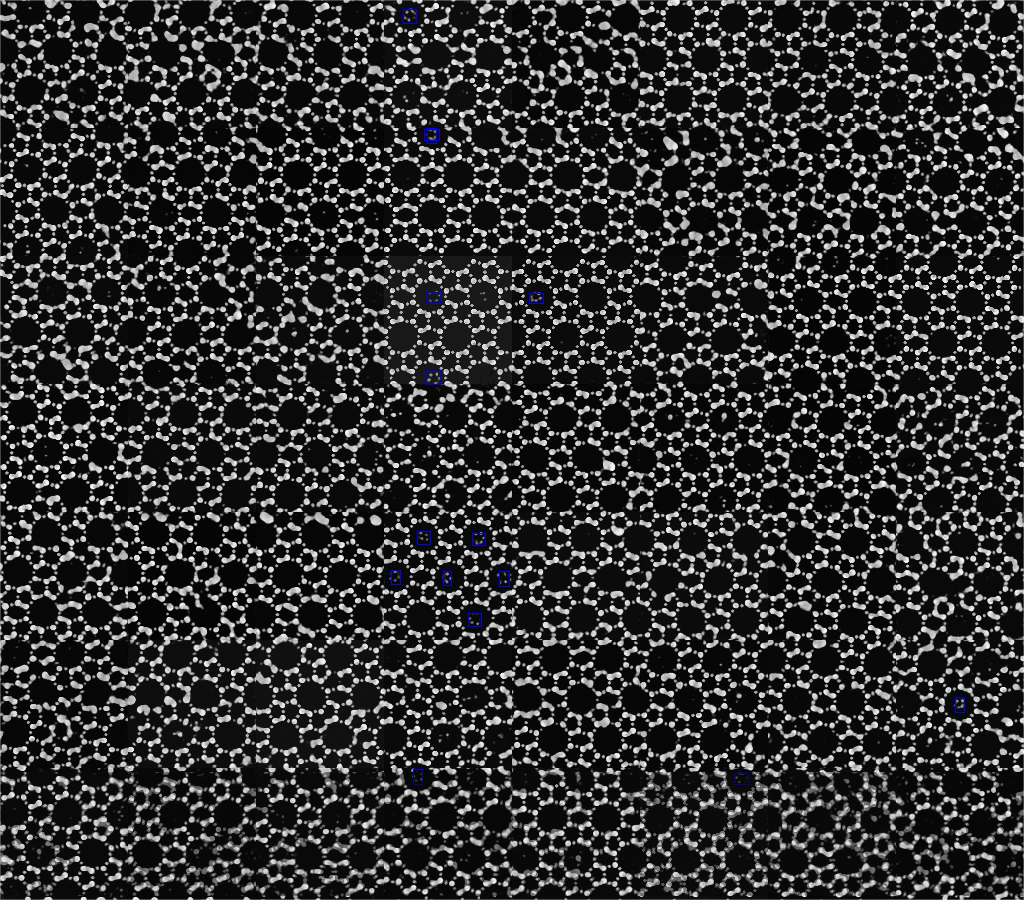
**

**Faster RCNN Detection Results**

**
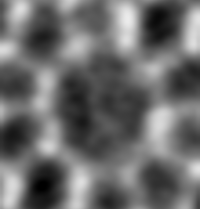
**

**Single-channel iDPC-STEM Images**

**
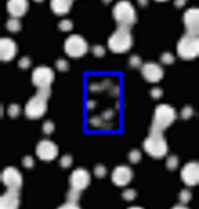
**

**Single-channel detection Result**

**Fig. S12.** A schematic of the CIF2Label program generating a label image.


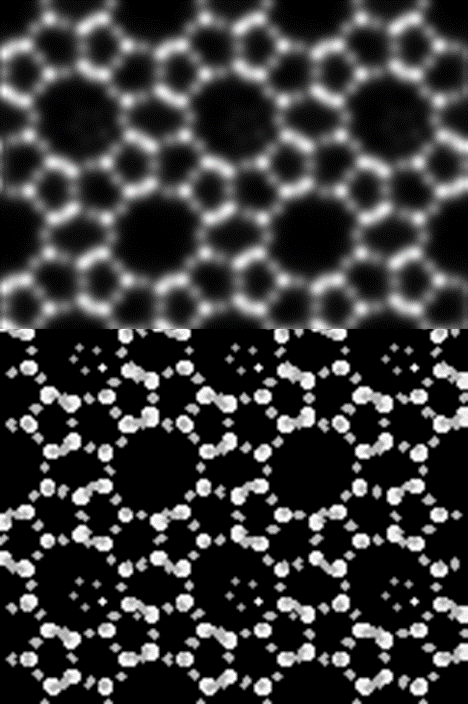

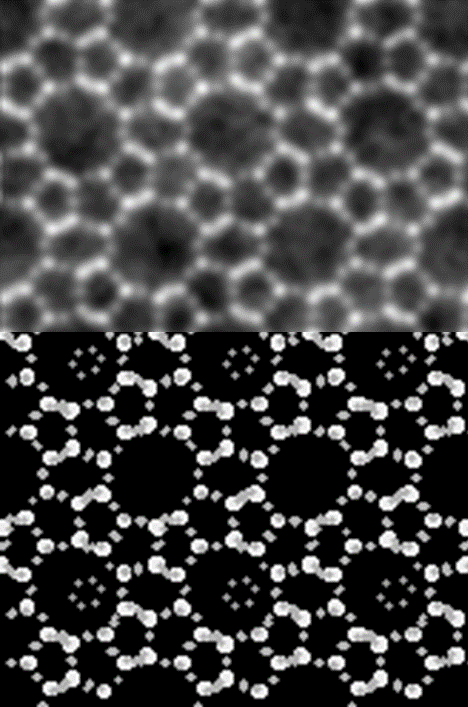


Simulated Thiophene Image Simulated Pyridine Image

**Fig. S13.** Detection of Molecular Presence and Vertical vs. Horizontal Orientations in Pores

**
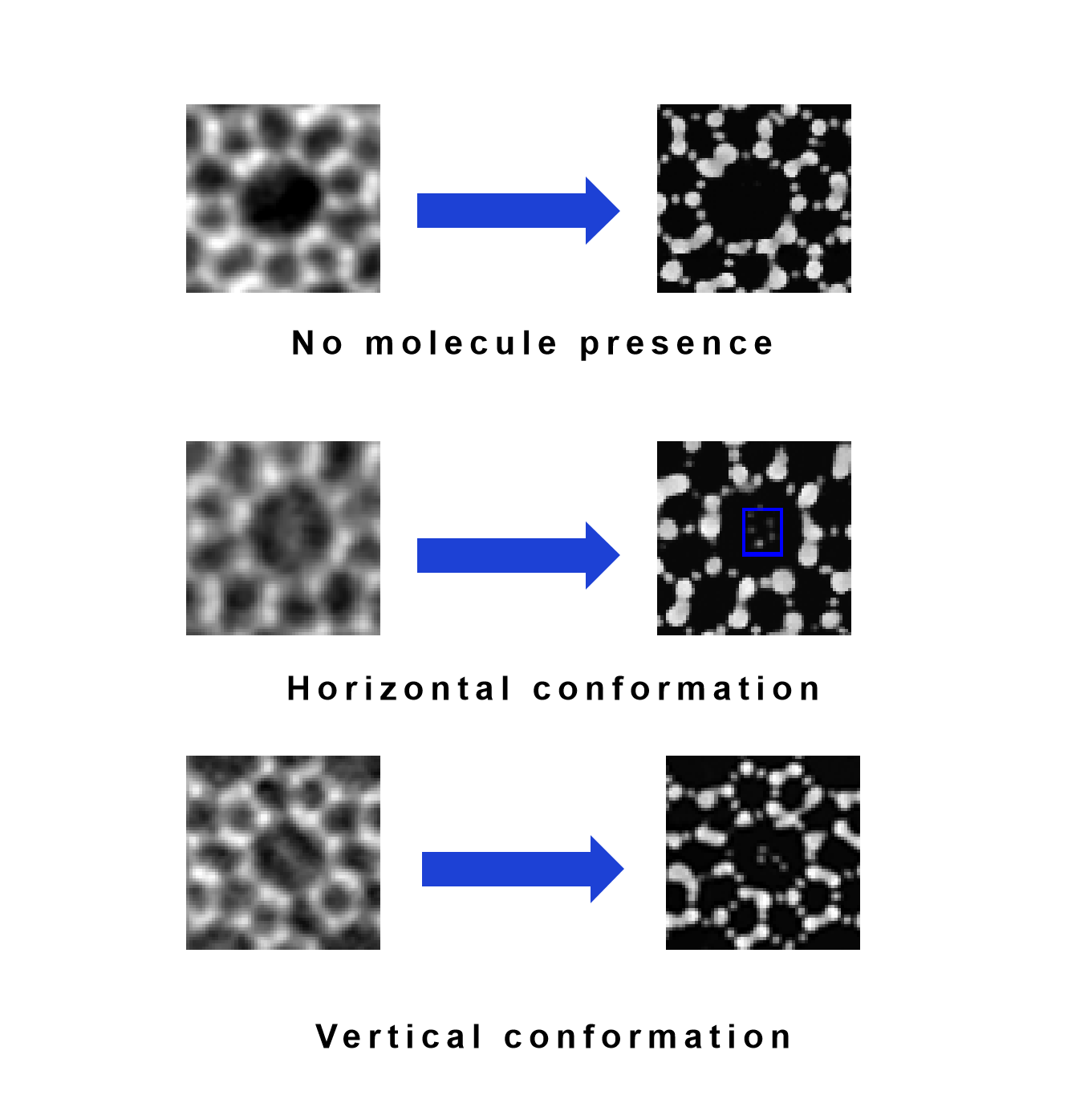
**

**Fig. S14.** Different model robustness and generalizability on real iDPC-STEM images.

**
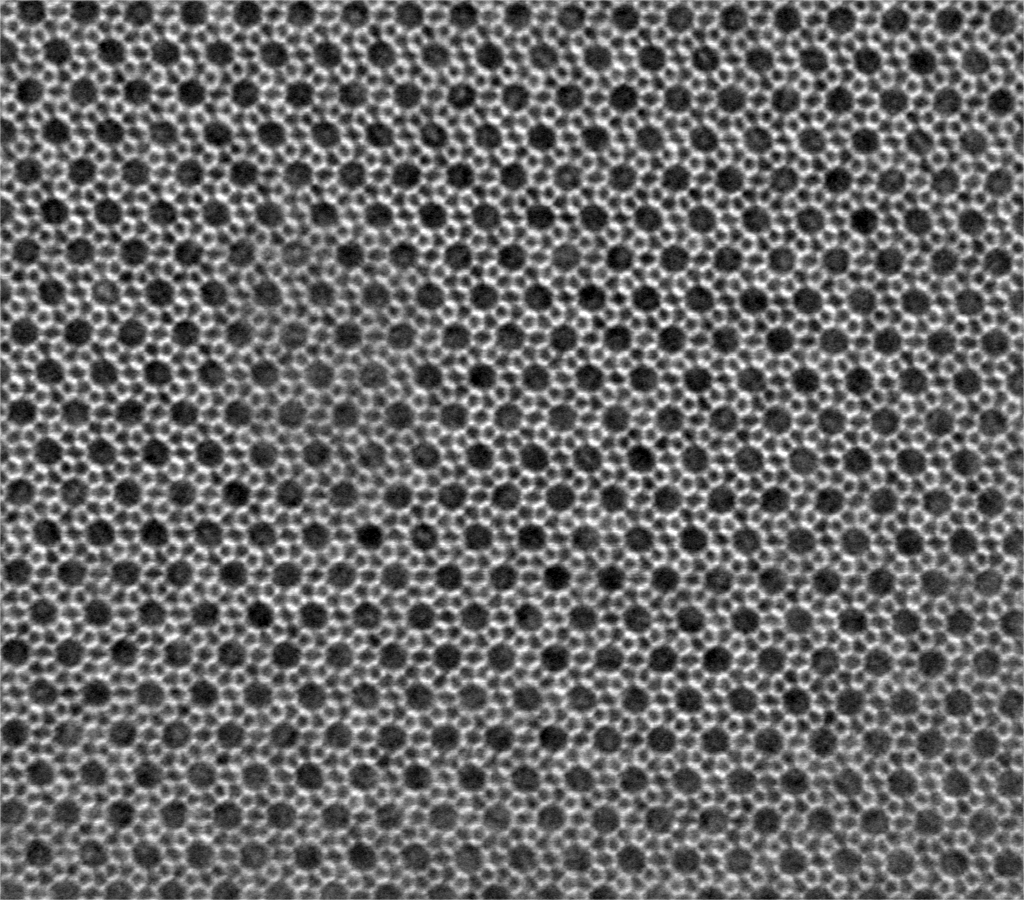
**

**Pyridine Real STEM Image**

**
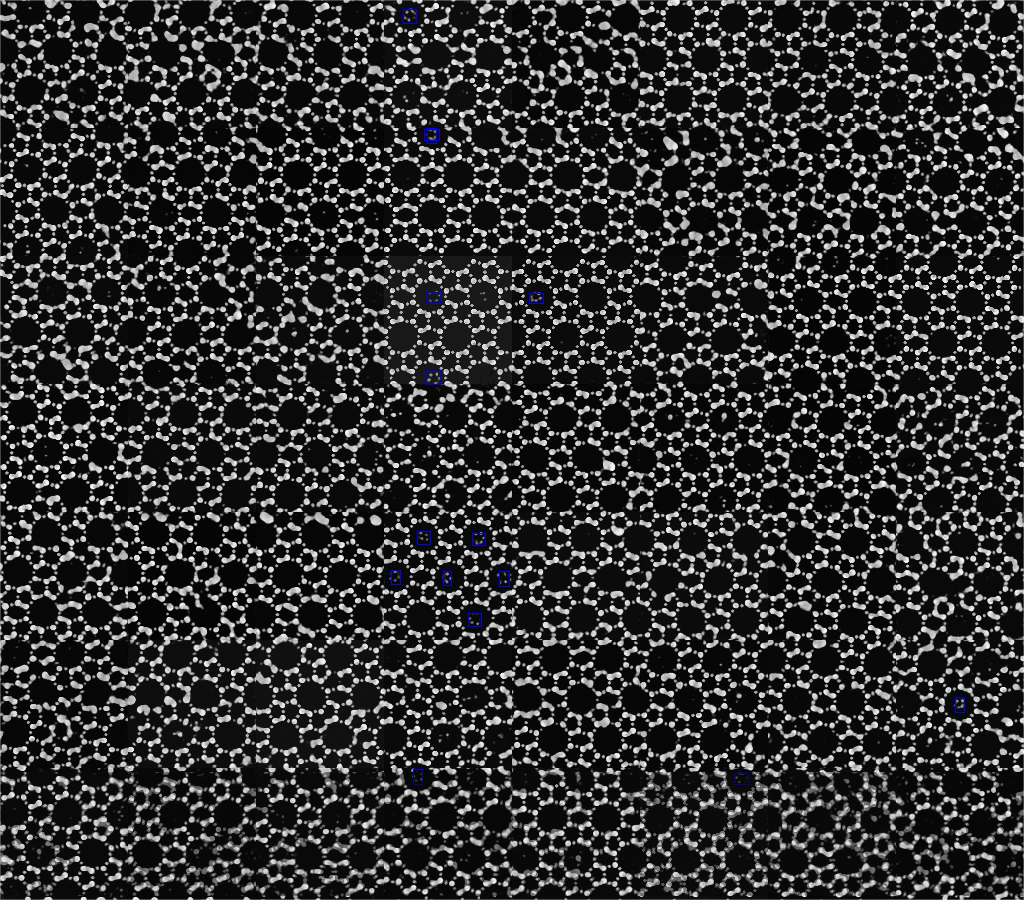
**

**DIVAESR Model Results**


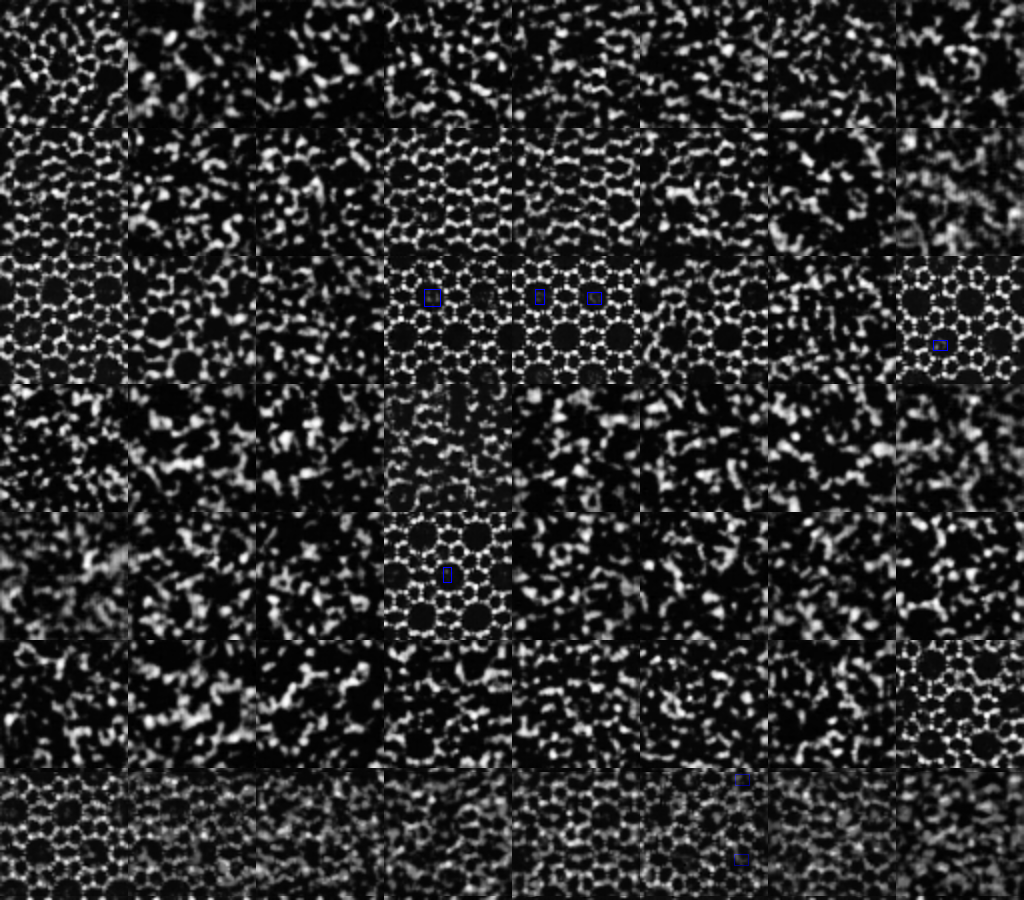


**VAE Model Results**


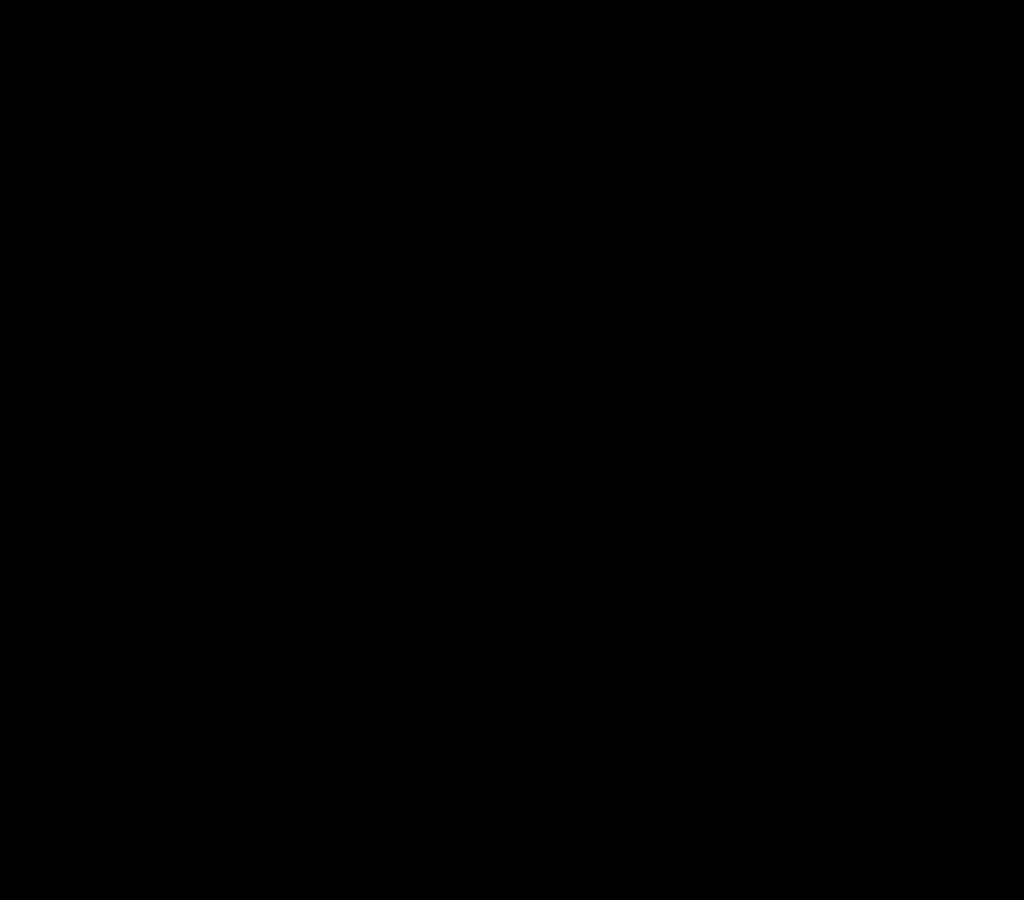


**SR Model Results**


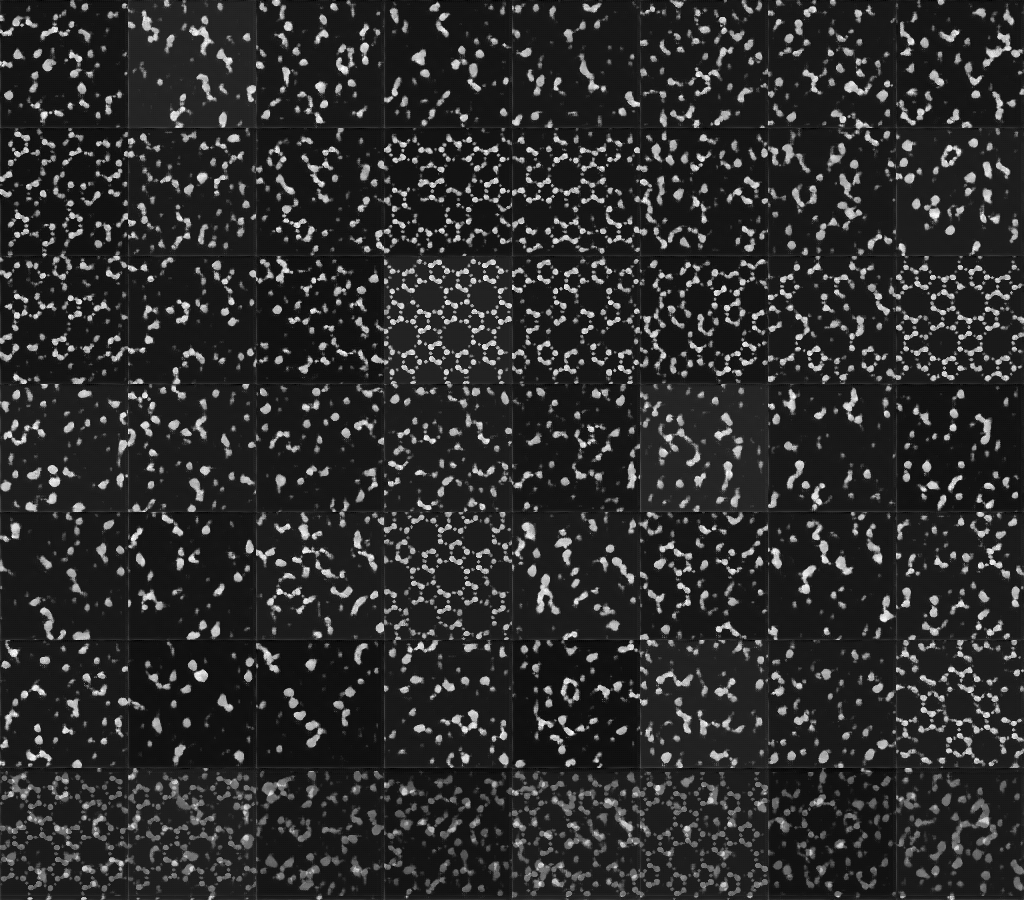


**VAESR Model Results**

**Table S1.** Determination of Optimal Hyper-parameter Combinations through Grid Search

| Train version index | Batch size | Learning rate | Epoch | MSE Error |
| --- | --- | --- | --- | --- |
| 1 | 64 | 0.0001 | 120 | 96.72 |
| 2 | 64 | 0.0005 | 120 | 58.05 |
| 3 | 64 | 0.001 | 120 | 51.68 |
| 4 | 64 | 0.005 | 120 | 103.35 |
| 5 | 128 | 0.0001 | 120 | 121.35 |
| 6 | 128 | 0.0005 | 120 | 70.99 |
| 7 | 128 | 0.001 | 120 | 65.19 |
| 8 | 128 | 0.005 | 120 | NA |
| 9 | 64 | 0.0001 | 90 | 97.68 |
| 10 | 64 | 0.0005 | 90 | 61.83 |
| 11 | 64 | 0.001 | 90 | 53.81 |
| 12 | 64 | 0.005 | 90 | NA |
| 13 | 128 | 0.0001 | 90 | 111.9 |
| 14 | 128 | 0.0005 | 90 | 82.21 |
| 15 | 128 | 0.001 | 90 | 73.5 |
| 16 | 128 | 0.005 | 90 | 486.14 |

Reference:

[1] aI. Lazić, E. G. Bosch, S. Lazar, *Ultramicroscopy* **2016**, *160*, 265-280; bJ. Madsen, T. Susi, *Microscopy and Microanalysis* **2020**, *26*, 448-450.

[2] B. Shen, H. Wang, H. Xiong, X. Chen, E. G. Bosch, I. Lazić, W. Qian, F. Wei, *Nature* **2022**, *607*, 703-707.

[3] aG. Kresse, J. Furthmüller, *Physical review B* **1996**, *54*, 11169; bJ. P. Perdew, K. Burke, M. Ernzerhof, *Physical review letters* **1996**, *77*, 3865.

[4] P. E. Blöchl, *Physical review B* **1994**, *50*, 17953.

[5] aS. Grimme, *Journal of computational chemistry* **2004**, *25*, 1463-1473; bS. Grimme, *Journal of computational chemistry* **2006**, *27*, 1787-1799.
